# The Role of the Surgical Pathologist in the Recognition of Hereditary Neuroendocrine Tumors

**DOI:** 10.1097/PAP.0000000000000551

**Published:** 2026-09-10

**Authors:** Silvia Uccella, Claudio Luchini, Atsuko Kasajima, Günter Klöppel, Stefano La Rosa

**Affiliations:** Departments of * Biomedical Sciences, Humanitas University, Milan; † Pathology Service, Istituti di Ricovero e Cura a Carattere Scientifico, Humanitas Research Hospital, Milan; ‡ Diagnostics and Public Health, Section of Pathology, University and Hospital Trust of Verona; § ARC-NET Research Center, University of Verona, Verona; ¶ Medicine and Technological Innovation, Unit of Pathology; # Medicine and Technological Innovation, Hereditary Cancer Research Center, University of Insubria; ** Unit of Pathology, University Hospital (ASST Sette Laghi), Varese, Italy; ∥ Pathology, TUM School of Medicine and Health, Technical University Munich, Munich, Germany

**Keywords:** hereditary tumor syndrome, neuroendocrine tumors, pituitary, parathyroid

## Abstract

About 10% of neuroendocrine tumors (NETs) can occur in the context of several different hereditary tumor syndromes, and, in a not negligible number of cases, their diagnosis precedes the clinical identification of the hereditary syndrome. In this context, the role of a pathologist can be crucial in identifying these sentinel cases. Indeed, early recognition of inherited NETs has significant clinical value, both for guiding therapeutic strategies and for managing at-risk family members. Modern pathologists play a central role not only in the accurate diagnosis and subtyping of NETs but also in recognizing specific morphologic features that may point to a germline etiology. When morphology does not show peculiar features suggestive of a hereditary context, integrating morphology with clinical context may, however, help identify appropriate genetic testing. In the present paper, we provide an organ-based overview of the most important morphologic and immunohistochemical features, along with their molecular correlates, of pituitary, parathyroid, thoracic, and digestive NETs arising in the context of hereditary tumor syndromes.

Epithelial neuroendocrine neoplasms (NENs) are ubiquitous neoplasms that may affect virtually any epithelium-containing organ. They include 3 categories: well-differentiated NENs, called neuroendocrine tumors (NETs); poorly differentiated NENs, defined as neuroendocrine carcinomas (NECs); and mixed neuroendocrine-non-neuroendocrine neoplasms (MiNENs).^1,2^ NECs and MiNENs are aggressive malignancies occurring sporadically and showing a molecular background similar to that of non-neuroendocrine carcinomas arising in the same sites.^3^ In turn, NETs, which are rather indolent and slow-growing neoplasms, are molecularly unrelated to the previous ones and reported to occur in the context of specific hereditary tumor syndromes in up to 10% of cases, as summarized in Table 1.^2,4,5^ Importantly, in a non-negligible number of these cases, the diagnosis of NET precedes the identification of the hereditary syndrome. In this context, the role of a pathologist can be crucial in identifying morphologic features suggestive of germline mutations and in recommending appropriate genetic testing, which serves as the starting point for clinical management.

**TABLE 1:** Hereditary Tumor Syndromes Associated With Epithelial Neuroendocrine Tumors (NETs)

Hereditary Syndrome	Gene	Chromosomal Locus	Tumor
MEN1 syndrome	*MEN1*	11q13.1	PitNET
			Parathyroid tumor
			Lung NET
			Thymic NET
			Gastric ECL-cell NET, type 2
			Duodenal NET (somatostatin and gastrin-producing)
			PanNET
MEN2A syndrome	*RET*	10q11.21	MTC
			Parathyroid tumor
MEN2B syndrome	*RET*	10q11.21	MTC
MEN4 syndrome	*CDKN1B*	12p13.1	Parathyroid tumor
			PitNET
			Thymic NET
			PanNET
MEN5 syndrome	*MAX*	14q23.3	PitNET
			Parathyroid tumor
Carney complex	*PRKAR1A*	17q24.2	PitNET
			Parathyroid tumor
Cowden syndrome	*PTEN*	10q23.31	Lung NET
McCune-Albright syndrome	*GNAS*	20q13	PitNET
DICER1 syndrome	*DICER1*	14q32.13	Pituitary blastoma
Neurofibromatosis type 1	*NF1*	17q11.2	PanNET
			Duodenal NET (somatostatin-producing)
			Rectal NET
Mavash disease	*GCGR*	17q25.3	PanNET (glucagon-producing)
Tuberous Sclerosis Complex	*TSC1*	9q34	PanNET
	*TSC2*	16p13	PanNET Rectal NET
Hyperparathyroidism-jaw tumor (HPT-JT) syndrome	*CDC73*	1q31.2	Parathyroid tumor
Von Hippel Lidau	*VHL*	3p25.3	PanNET
Familial insulinomatosis	*MAFA*	8q24.3	PanNET (insulin-producing)
SDH-deficient tumor syndromes	*SDHD*	11q23	PitNET
	*SDHAF2*	11q12.2	PitNET
	*SDHC*	1p23.3	PitNET
	*SDHB*	1p36.1-p35	PitNET
	*SDHA*	5p15	PitNET

MTC indicates medullary thyroid carcinoma; NET, neuroendocrine tumor; PanNET, pancreatic neuroendocrine tumor; PitNET, pituitary neuroendocrine tumor.

In the present paper, we provide an organ-based overview of the most important morphologic and immunohistochemical features, along with their molecular correlates, of NETs arising in the context of hereditary tumor syndromes. Medullary thyroid carcinoma, which is in fact a NEN, is not treated here, as it is the subject of a specific article in this special issue.

## PITUITARY GLAND

Pituitary neuroendocrine tumors (PitNETs) are a wide group of anterior pituitary neuroendocrine tumors encompassing several different clinicopathological entities. The current classification of PitNETs is based on cell lineage identification through the expression of specific transcription factors (PIT1, TPIT, and SF1). Subtyping relies on the combination of cell morphology, hormone secretion (GH, PRL, ACTH, FSH, LH, and TSH), and other features (including, but not limited to, cytokeratins expression patterns and other transcription factors expression).^6^ While the vast majority of PitNETs are sporadic with unclear molecular pathogenesis, about 5% arise in the context of familial setting, either isolated (familial isolated pituitary adenoma or FIPA) or as part of a hereditary tumor syndrome (Fig. 1). These cases need to be identified, as they may represent the “index case” in a family with unknown hereditary predisposition and, importantly, they tend to show a more aggressive clinical behavior.^7^

**FIGURE 1:**
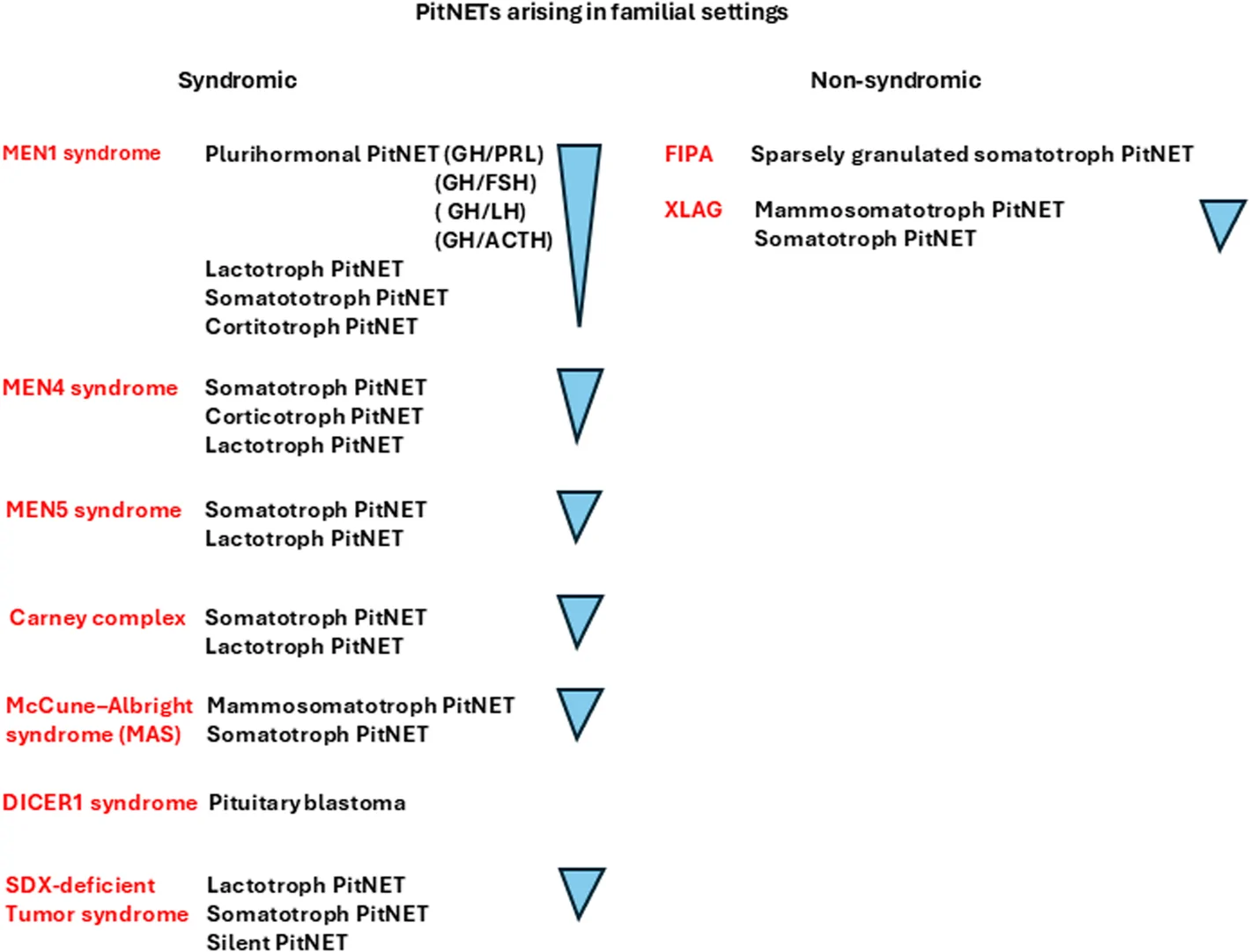
Frequency of pituitary neuroendocrine tumors (PitNETs) in syndromic and nonsyndromic familial settings. FIPA indicates familial isolated pituitary adenoma; XLAG: Χ-linked acrogigantism.

The most common hereditary syndromes in which PitNETs may appear are multiple endocrine neoplasia type 1 (MEN1), multiple endocrine neoplasia type 4 (MEN4), Carney complex, and DICER1 syndrome. These and other, less frequent, conditions are listed below and summarized in Box 1.

BOX 1 Features of Hereditary PitNETs

**Table:** 

Syndrome	Typical PitNET Pattern	Key Pathologic/Clinical Clues	Main Gene
MEN1	Often larger, invasive, and sometimes plurihormonal; common lineages include lactotroph and somatotroph.	Young age, recurrence, associated hyperparathyroidism, or pancreatic NETs; the morphology usually overlaps with that of sporadic tumors.	MEN1
MEN4	Similar to MEN1 but rarer; PitNETs are often somatotroph or corticotroph.	Consider when MEN1-like phenotype but MEN1-negative; no specific morphologic signature.	CDKN1B
MEN5	Pit1-lineage, especially somatotroph/lactotroph.	Morphology is not distinctive; MAX IHC has limited routine value.	MAX
Carney complex	Usually Pit1-lineage, often somatotroph or mammosomatotroph.	Hyperplasia of somatotroph/lactotroph cells may precede a tumor; it tends to be hormonally subtle at first.	PRKAR1A
DICER1 syndrome	Pituitary blastoma rather than conventional PitNET.	Infants/very young children, severe Cushing syndrome; biphasic/triphasic primitive morphology.	DICER1
SDH-deficient syndrome	Usually large, aggressive PitNETs, often lactotroph or somatotroph.	Intracytoplasmic vacuoles may be a clue; loss of SDHB supports the diagnosis.	SDHx/SDHAF2
FIPA/AIP	Usually, sparsely granulated somatotroph tumors.	Young patients, macroadenomas, invasive behavior; no specific histology.	AIP
XLAG	Mixed somatotroph-lactotroph tumor or hyperplasia.	Very early-onset gigantism, often in girls, with sinusoidal/lobular architecture and calcifications.	GPR101

### Multiple Endocrine Neoplasia Type 1 Syndrome

MEN1 syndrome is an autosomal-dominant hereditary syndrome affecting patients of a wide range of ages (from 5 to 81 y). Clinical symptoms and/or biochemical abnormalities present more frequently in the fifth decade of life, generally after the age of 21. Only a minority of MEN1 patients (about 14%) develop symptoms before age 10 years.^8^ Primary hyperparathyroidism due to parathyroid tumors (single or multiple adenomas or carcinomas) is the most common clinical presentation (in about 95% of MEN1 patients), followed by pancreatic NETs (40% of patients), and PitNETs (30% of patients). In addition, other associated endocrine and nonendocrine neoplasms can be observed, including adrenal cortical tumors (40%), pheochromocytoma (<1%), lung NETs (2%), thymic NETs (2%), gastric NETs (10%), lipomas (30%), angiofibromas (85%), and meningiomas (8%).^9^

MEN1 syndrome is caused by mutations in the *MEN1* gene (located on chromosome 11q13), which encodes MENIN, a protein involved in several cellular processes, including transcriptional regulation, genome stability, cell division, and proliferation.^9^ About 460 different mutations scattered throughout the entire coding region and splice sites of the *MEN1* gene have been identified (nonsense mutations, frameshift deletions or insertions, in-frame deletions or insertions, splice-site mutations, and missense mutations). A minority of patients with MEN1 syndrome do not harbor mutations in the coding region but instead show large gene deletions or promoter mutations.^9^ As a whole, mutations of the *MEN1* gene determine a decrease in MENIN level and activity, which in turn results in a decrease of genome stability, loss of suppression of multiple transcription complexes, and a decrease of P27 function with consequent loss of inhibitory control on proliferation. Notably, patients with *MEN1* gene germline mutations are at risk of developing a somatic mutation or deletion of the second allele later in life. The timing of this somatic alteration may explain the variability of ages at diagnosis.^10^

MEN1-associated PitNETs are usually diagnosed before the age of 50 years or older, and patients tend to be younger than those with sporadic PitNETs.^11,12^ Women are more frequently affected than men.^13^ Tumors are commonly large, locally invasive, and are at high risk of recurrence after therapy.^8^ Almost 40% of MEN1-related PitNETs are reported to secrete multiple hormones. Indeed, plurihormonality with co-secretion of GH and prolactin, and unusual associations with FSH, LH, or ACTH, are more frequent in MEN1-associated than in sporadic PitNETs. Other PitNET subtypes encountered in MEN1 syndrome are lactotroph (20%), somatotroph (10%), corticotroph (<5%), and nonfunctioning (silent) PitNETs (<5%) (Fig. 2).^12,13^

**FIGURE 2:**
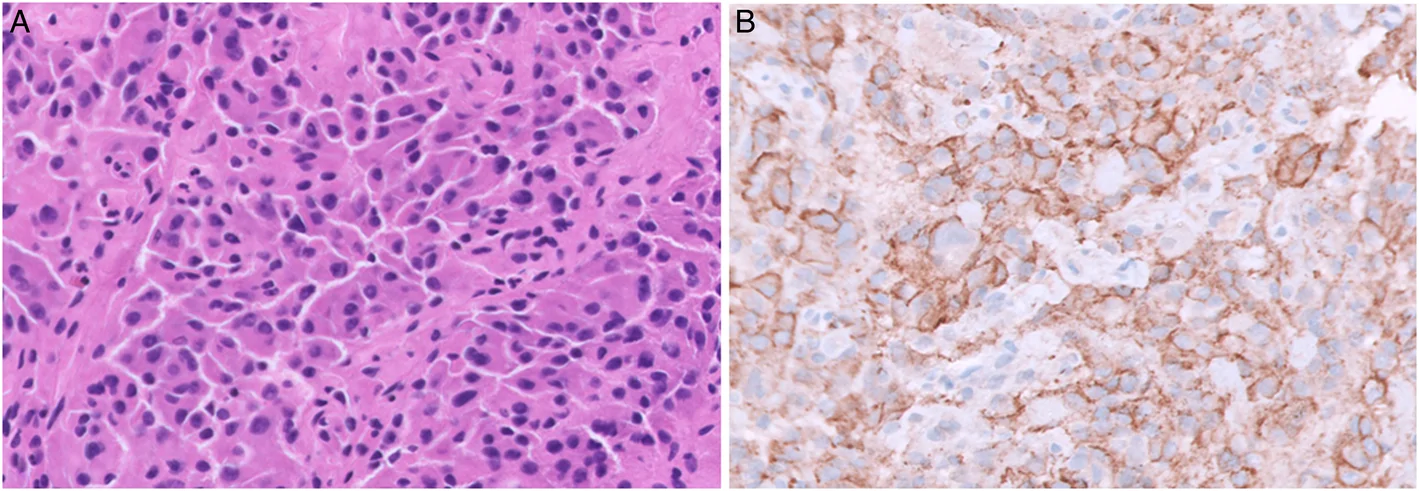
Sparsely granulated lactotroph PitNET in a patient with MEN1 syndrome composed of oxyphil cells (A) immunoreactive for prolactin (B).

The morphology of MEN1-associated PitNETs overlaps with that of sporadic ones, and, unfortunately, there are no histopathologic features useful to distinguish them from sporadic PitNETs,^14^ except for the apparent increased immunoreactivity for S100 protein in folliculo-stellate cells.^13^ The role of MENIN immunohistochemistry in identifying MEN1-associated PitNETs is not demonstrated, since MENIN expression was found to be significantly reduced in a high percentage of sporadic PitNETs.^15^ In fact, pathologists can only integrate their diagnosis with additional clinico-pathologic features to suggest a possible association with the MEN1 syndrome. In this context, the recommendations of the European Society of Endocrinology suggest performing genetic testing (to search for pathogenic variants in MEN1 and/or AIP) in patients with aggressive PitNETs, considering patients' age at diagnosis and a positive family history of pituitary or neuroendocrine neoplasms.^16^

In general, genetic tests to identify *MEN1* gene mutations should be performed in patients with:

- PitNET associated with
  - positive family history of pancreatic, pituitary, and parathyroid tumors;
  - young age and hormonally active tumor;
  - hypercalcemia or other endocrinopathies;
- Zollinger-Ellison syndrome;
- Multiple pancreatic NETs;
- Hyperparathyroidism and multiple parathyroid adenomas (especially if younger than 45 y).^17–19^

Since the family history may be uninformative because affected family members may have died before endocrine symptoms develop, pathologists play a crucial role in identifying suspected cases. From a practical standpoint, when evaluating PitNETs, especially Pit1-lineage tumors with locally aggressive behavior arising in young patients, it is strongly recommended to add a comment in the pathology report suggesting genetic testing.

### Multiple Endocrine Neoplasia Type 4 Syndrome

A small percentage of patients with clinical features of MEN1 syndrome do not show *MEN1* gene mutations but instead have frameshift, nonsense, missense, or 5′ UTR mutations in the *CDKN1B* gene. This condition is known as MEN4 syndrome and is inherited in an autosomal dominant pattern.^20^ *CDKN1B* is located on chromosome 12q13 and encodes for p27, a member of the cyclin-dependent kinase inhibitors family, which negatively regulates the cyclin E/cyclin-dependent kinase 2 complex, inhibiting the cell cycle.

After parathyroid adenomas, PitNETs are the second most common tumor type in MEN4 patients, comprising somatotroph (10% of MEN4 patients), corticotroph (5%), and, more rarely, lactotroph PitNETs.^21–23^ Digestive NETs are observed in about 25% of MEN4 patients.^24^ Since a limited number of cases have been reported in the literature, there is no detailed information on the morphology of MEN4-related tumors that, however, appear to show the same morphologic features of corresponding sporadic tumors. P27^Kip1^ immunohistochemistry may be of help in detecting MEN4-related neoplasms. However, it is worth noting that P27^Kip1^ protein expression is reduced during the progression from normal to neoplastic pituitary tissues, as well as in other neuroendocrine tumors, including medullary thyroid carcinoma, parathyroid adenomas, and gastrointestinal and pancreatic NENs.^25^ For these reasons, P27^Kip1^ immunohistochemistry should be used with caution and interpreted in the clinical context. For practical and clinical purposes, the possibility of MEN4 syndrome should be explored when a patient with clinical features of MEN1 syndrome does not harbor *MEN1* gene mutations.^24,25^

### Multiple Endocrine Neoplasia Type 5 Syndrome

MEN5 syndrome is an inherited tumor syndrome caused by pathogenic germline sequence variants in the tumor suppressor gene *MAX* (14q23.3), which encodes the MYC-associated protein X (MAX), an essential component of the MYC/MAX/MXD1 network of transcription factors that regulate cell proliferation, differentiation, and apoptosis.^26,27^ Germline nonsense and missense mutations and large deletions of *MAX* result in a modified protein unable to repress MYC signaling.^26,28^

MAX-associated PitNETs, histologically indistinguishable from their sporadic counterparts, are of Pit1 lineage with somatotroph or lactotroph cell differentiation.^29–31^ Since morphology is not useful for identifying PitNETs in the setting of MEN5 syndrome, some studies have investigated the possible role of MAX immunohistochemistry. However, although loss of MAX immunoreactivity seems to occur only in MAX-related tumors,^32,33^ the reported sensitivity and specificity as a marker of *MAX* mutation is poor.^30,34^ For this reason, MAX immunohistochemistry in routine clinical practice appears to be of limited value and is currently not recommended as a screening test for *MAX* mutation.^30^

### Carney Complex

The Carney complex is an autosomal dominant tumor predisposition syndrome characterized by the presence of spotty skin pigmentation, myxomatosis, endocrine tumors (pigmented nodular adrenocortical disease, PitNETs, and thyroid tumors), breast duct adenoma, testicular and ovarian tumors, and pigmented schwannoma. It is related to mutations in the *PRKAR1A* gene, located on 17q, which encodes the type 1α regulatory subunit of PKA, known as an important effector molecule in many endocrine signaling pathways.^35^ In addition, the *PRKAR1A* gene seems to act as a tumor suppressor gene, and its mutations determine the loss of function of the PRKAR1A protein, which leads to constitutive activation of PKA with enhanced of its intracellular signaling.^36^

Regarding PitNETs, about 75% of patients present an asymptomatic increase of GH and IGF-1 levels. Subtle hyperprolactinemia is detected in about 65% of cases, while only 12% tend to develop clinical symptoms of acromegaly.^35,37^

Hyperplasia of somatotroph and lactotroph cells may precede the development of PitNETs, which may explain the insidious and protracted period before the identification of clinical endocrine symptoms.^38^ Once developed, PitNETs show the same morphologic features of their sporadic counterparts. Most PitNETs are of Pit1-lineage with somatotroph or mammosomatotroph cell differentiation, often with densely granulated tumor cells (Fig. 3).^39^

**FIGURE 3:**
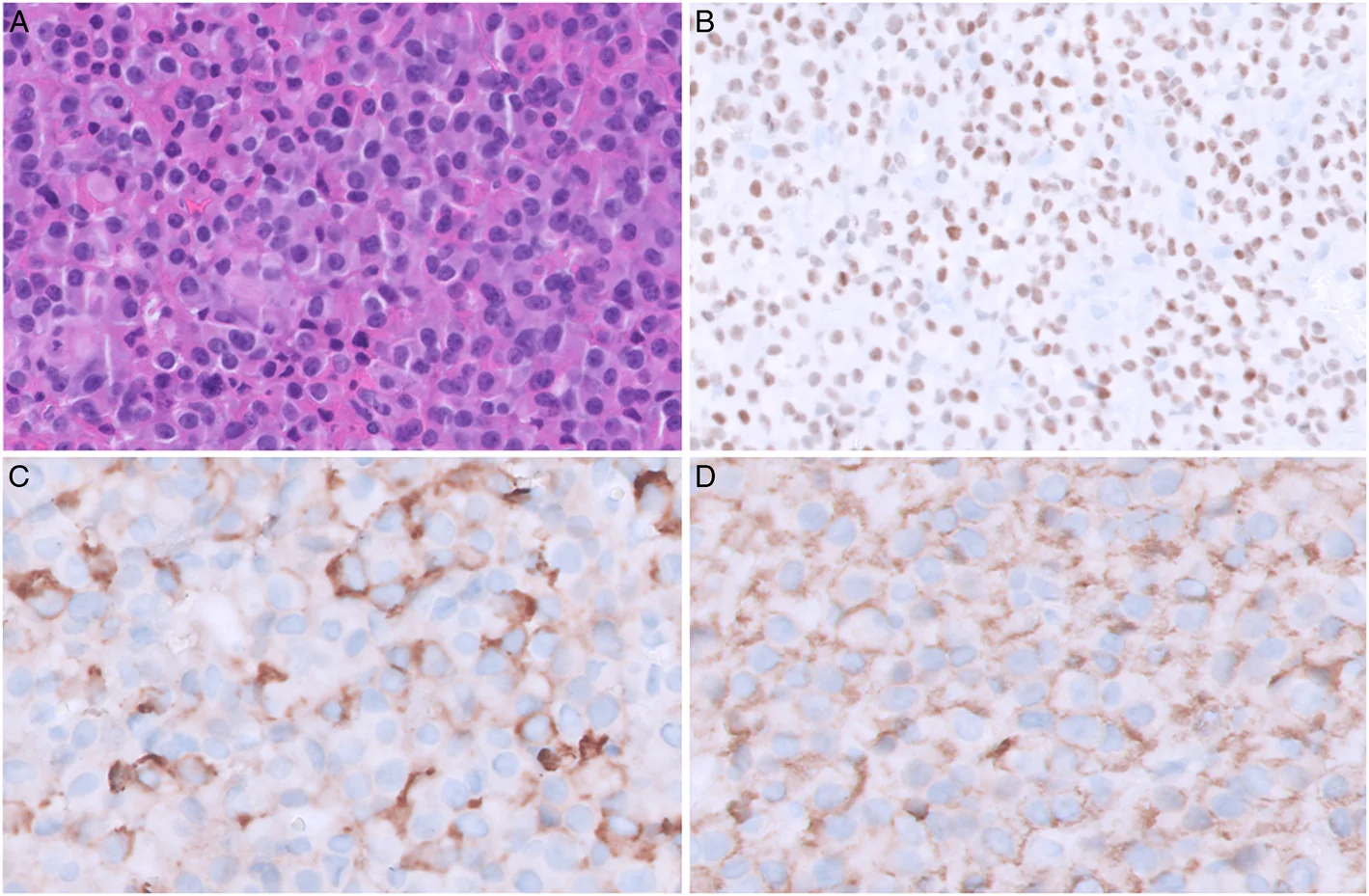
Example of mammosomatotroph PitNET composed of monomorphic cells (A) positive for Pit-1 (B). In addition, tumor cells are positive for both prolactin (C) and growth hormone (D).

### McCune-Albright Syndrome

McCune-Albright syndrome (MAS) is rare, with an estimated prevalence ranging between 1 case per 100,000 and 1 million people.^40^ The clinical presentation is characterized by a wide clinical variability with a broad spectrum of endocrine and nonendocrine manifestations, including the classic triad of polyostotic fibrous dysplasia, precocious puberty (especially in females), and skin hyperpigmentation (café-au-lait skin macules). Additional endocrine manifestations include hyperthyroidism, testicular lesions (Leydig and/or Sertoli cell hyperplasia), GH excess, and, more rarely, neonatal hypercortisolism. GH excess is observed in about 20% of patients and, in most cases, is associated with hyperprolactinemia due to PitNET (30% to 50% of cases) or pituitary hyperplasia.^41,42^

MAS is characterized by a somatic mosaicism due to activating mutations of either residue Arg201 or, rarely, Gln227 of the *GNAS* gene (20q13.32) that occur at early postzygotic stage.^43^ *GNAS* gene encodes the cAMP pathway associated G protein Gs-alpha and mutations cause loss of the GTPase activity with consequent permanent activation of the adenylate cyclase and constitutive activation of the cAMP dependent PKA pathway, which induces increased cell proliferation in cAMP-responsive tissue, such as the pituitary gland.

### DICER1 Syndrome

DICER1 syndrome is an autosomal dominant predisposition syndrome with low penetrance caused by heterozygous germline pathogenic variants in the DICER1 gene, with a prevalence in the general population of 1 in 10,600 people.^44,45^ Affected patients may develop different benign and malignant neoplasms involving endocrine organs (ie, thyroid and pituitary) or nonendocrine organs/system such as the lung (pleuropulmonary blastoma), kidney (cystic nephroma), female genital tract (embryonal rhabdomyosarcoma of the uterine cervix, ovarian Sertoli-Leydig cell tumor, and gynandroblastoma), and other sites including CNS, upper respiratory tract, gastrointestinal system, and soft tissues.^44^ *DICER1* gene, located on chromosome 14q32.13, encodes the DICER protein, which is a ribonuclease (RNase) III endoribonuclease essential to produce microRNAs (miRNAs) playing a crucial role in the control of protein translation.^46^

Pituitary blastoma is the most common lesion in the pituitary gland in this syndrome, although it is exceedingly rare (<1% of patients with DICER1 syndrome). Pituitary blastoma is an aggressive tumor usually arising in young children (median age at presentation is 8 mo with a range from 7 to 24 mo), generally presenting with severe Cushing syndrome, sometimes associated with diabetes insipidus, ophthalmoplegia, and fatal behavior in about 40% of the cases.^7,47,48^ These tumors are composed of 3 different types of cells: small undifferentiated blastemal cells, large adenohypophysial neuroendocrine cells arranged in lobules or diffuse sheets, and cuboidal or columnar primitive Rathke pouch epithelium forming rosette or gland/follicle formation (Fig. 4).^48^ A case of aggressive pituitary blastoma lacking Cushing syndrome and associated with hyperprolactinemia has recently been described.^49^

**FIGURE 4:**
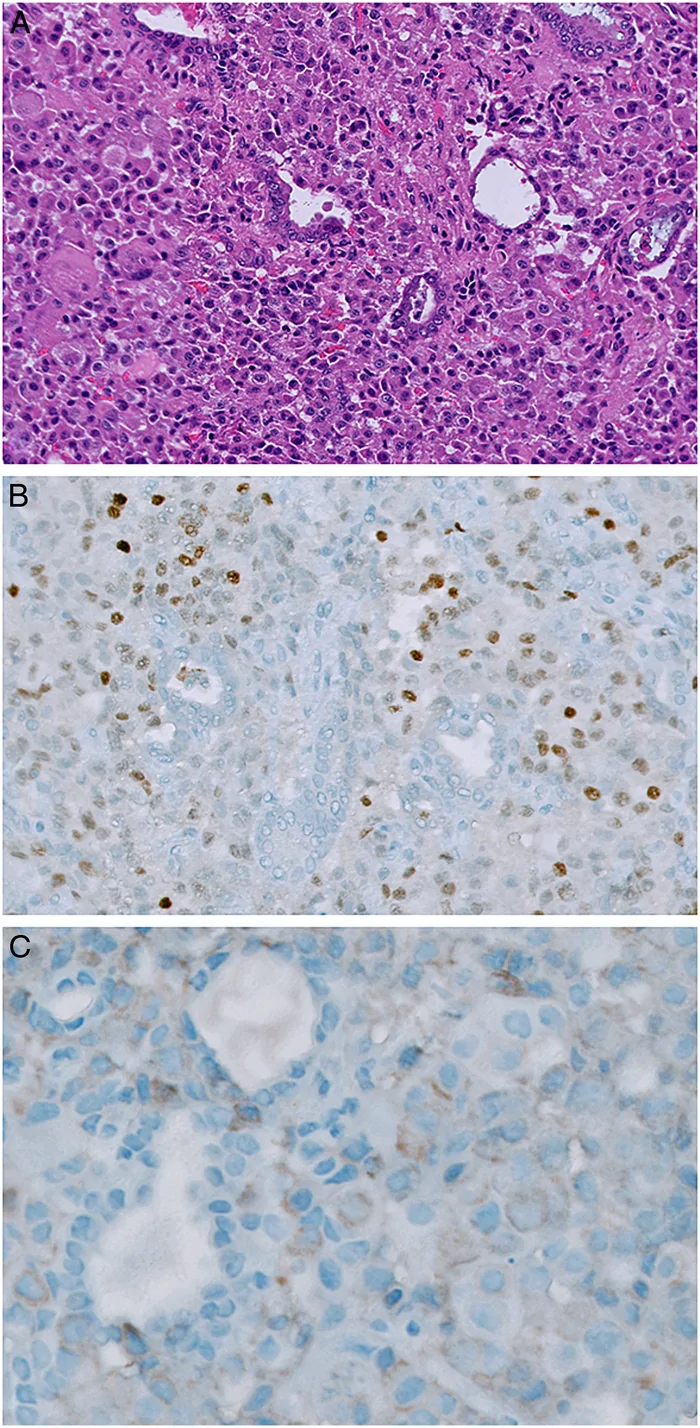
Example of pituitary blastoma composed of adenohypophysial neuroendocrine cells growing in a diffuse sheet, columnar primitive Rathke pouch epithelium forming “gland-like” structures and scattered small undifferentiated blastemal cells (A). Adenohypophyseal cells are positive for TPIT (B) and show a weak immunoreactivity for ACTH (C).

### SDH-Deficient Tumor Syndrome (Hereditary Phaeochromocytoma-Paraganglioma Syndrome)

The so-called succinate dehydrogenase (SDH)-deficient tumor syndrome, firstly described in patients with hereditary paraganglioma (PPGL),^50^ actually includes a group of syndromic diseases characterized by SDH-deficient neoplasm. These neoplasms are associated with either germline pathogenic mutations in one of the *SDH* genes encoding the subunits of the SDH complex (SDHA, SDHB, SDHC, SDHD, and SDHAF2) or promoter hypermethylation of the *SDHC* gene.^51^ Although SDH-deficient tumor syndromes show overlapping features, there is a genotype-phenotype correlation associating specific *SDH* gene alterations with certain tumors and sites (Table 2). In decreasing order, neoplasms that can be found in these syndromes are intra- and extra-adrenal paraganglioma, gastrointestinal stromal tumor (GIST), renal cell carcinoma, PitNET, and pulmonary chondroma.^52^

**TABLE 2:** SDH-Deficient Tumor Syndrome (Hereditary Pheochromocytoma-Paraganglioma Syndrome)

Syndrome	Gene	Chromosomal Locus	Pheochromocytoma (Adrenal PG)	Abdominal PG	Thoracic PG	H and N PG	GIST	RCC	PitNET
PGL1	SDHD	11q23.1	+	++	+	frequent	+	rare	+
PGL2	SDHAF2	11q12.2	rare	rare	rare	+	rare	rare	+
PGL3	SDHC	1q23.3	rare	rare	+	++	+	++	+
PGL4	SDHB	1p36.13	+	frequent	++	+	+	++	+
PGL5	SDHA	5p15.33	+	++	rare	+	frequent		++

GIST indicates gastrointestinal stromal tumor, SDH-deficient; H and N, head and neck; PG, paraganglioma; PitNET, pituitary neuroendocrine tumors; RCC, renal cell carcinoma, SDH-deficient.

SDH is a mitochondrial enzyme composed of 5 protein subunits: the anchoring components SDHC and SDHD, the catalytic components SDHA and SDHB, and SDHAF2 required for flavination of the SDHA protein. If any component of the SDH complex is lost or dysfunctional, the entire SDH complex becomes unstable, and the SDHB subunit is released into the cytoplasm, where it is rapidly degraded.^53,54^ The biological consequence of SDH dysfunction is the accumulation of succinate, which acts as an oncometabolite involved in several tumorigenic pathways, including hypoxic regulation and epigenetic reprogramming.^55,56^

Although the association between adrenal paraganglioma (pheochromocytoma) and somatotroph PitNET was described about 70 years ago,^57^ the causative genetic link between these 2 neuroendocrine neoplasms was understood only in 2012 when Xekouki and coworkers described a patient bearing a germline *SDHD* mutation in exon 3 (c.298_301delACTC) who presented bilateral adrenal paragangliomas (pheochromocytomas) and a concomitant somatotroph PitNET. This PitNET showed LOH at the *SDHD* locus and reduced expression of both SDHD and SDHB proteins and increased hypoxia-inducible factor 1α (HIF-1α) levels.^58^

SDH-deficient PitNETs are particularly rare, representing 0.3% of PitNETs in a series of unselected tumors.^59^ The most frequent SDH-deficient PitNET type is lactotroph, followed by somatotroph and nonfunctioning PitNET. These tumors tend to be large and clinically aggressive^52,60,61^ and a case of metastatic PitNET has been described.^62^ Generally, SDH-deficient PitNETs show the typical architecture and morphology of non-SDH-deficient PitNETs,^52,59–61,63^ although the presence of intracytoplasmic vacuoles seems characteristic and should alert the pathologist to a possible SDH syndrome.^62,64,65^ Nevertheless, given the extreme rarity of SDH-deficient PitNETs, routine immunohistochemical screening for SDHB protein loss or genetic screening for SDH mutations is not justified unless a possible SDH syndrome is suggested by the family history.^59^ Considering the aggressive phenotype of SDH-deficient PitNETs and the potential risk of metastatic dissemination, all patients known to carry mutations in one of the *SDH* genes should be endocrinologically and radiologically screened for PitNETs, although the incidence of these tumors is very low.^43^

SDHB immunohistochemistry is a useful tool for identifying tumors arising in the context of an SDH-deficient syndrome.^66^ SDHB immunostaining in SDH-proficient cells shows a granular cytoplasmic pattern (“mitochondrial pattern”), and the global loss of SDHB expression is associated with mutation of genes encoding for the SDH complex.^67^ As virtually all *SDH* genes mutations are germline, the absence of SDHB immunohistochemical stain is consistently associated to SDH-deficient syndrome.^67^ Practicing pathologists should interpret SDHB immunostaining with caution. Immunoreactivity for SDHB is considered true when neoplastic cells are negative, and the internal control (endothelial cells) is positive. It is worth noting that aberrant cytoplasmic SDHB staining, which can be misinterpreted as “retained,” has been described and represents a possible diagnostic pitfall.^68^ Aberrant cytoplasmic expression in tumor cells includes both weak diffuse cytoplasmic blushes lacking definite granularity and abnormal intracytoplasmic distribution, such as globular perinuclear accentuation.^67,68^ In most of these cases, after careful examination of the entire tumor, convincing areas of complete loss are identified.^68^

### Familial Isolated Pituitary Adenoma

FIPA was described in the late 1990s as the familial occurrence of PitNETs outside of the setting of syndromic conditions such as MEN1 syndrome and Carney complex.^69^ FIPA comprises ∼2% of PitNETs and is an autosomal dominant disease with incomplete penetrance in which PitNETs occur in 2 or more related family members in the absence of known familial or genetic syndromes.^7^

Most FIPA cases have no known genetic cause, although a germline mutation in the aryl hydrocarbon receptor-interacting protein (*AIP*) gene, located on chromosome 11q13, is detected in about 20% of FIPA patients.^70,71^ The *AIP* gene encodes a 330-amino-acid protein^72^ related to secretory granules in lactotroph and somatotroph cells.^7,72^ Most *AIP* gene mutations are nonsense, missense, frameshift, splicing, and promoter mutations resulting in premature stop codons, while less than 10% of genetic alterations are large deletions.^43^

The exact mechanisms through which *AIP* mutations induce pituitary tumorigenesis are not completely understood, but mutation in *AIP* gene is known to (1) alter the normal function of aryl hydrocarbon receptor (AhR) with consequent alteration of normal cell cycle regulation;^73^ and (2) block the unliganded RET-pathway with consequent promotion of GH cells survival, leading to GH-cell hyperplasia and PitNET,^74^ and stimulation of PKA activity, known as a predisposing factor for pituitary tumorigenesis.^75^ Thus, patients with *AIP* mutations are predisposed to early development of large PitNETs with aggressive features, such as local invasion.^76–78^ In most cases, patients develop acromegaly-associated somatotroph PitNETs, followed by lactotroph tumors and, more rarely, nonfunctioning PitNETs.^7^ It is worth noting that somatotroph PitNETs in patients with *AIP* mutations show low expression of somatostatin receptor 2, being less sensitive to treatment with somatostatin analogs such as lanreotide.^79^

Histologically, PitNETs arising in the context of FIPA are more frequently sparsely granulated somatotroph tumors (Fig. 5) but do not show specific FIPA-associated morphologic features. For this reason, pathologists lack diagnostic morphologic tools for identification. In addition, AIP immunohistochemical expression is not useful for identifying FIPA patients, as it is reduced or negative also in sporadic somatotroph PitNETs, especially if sparsely granulated or treated with somatostatin analogs.^80^ However, pathologists can make the clinicians aware of a possible association with FIPA when encountering sparsely granulated somatotroph PitNETs in adolescents or children, especially if the tumor shows local invasiveness.

**FIGURE 5:**
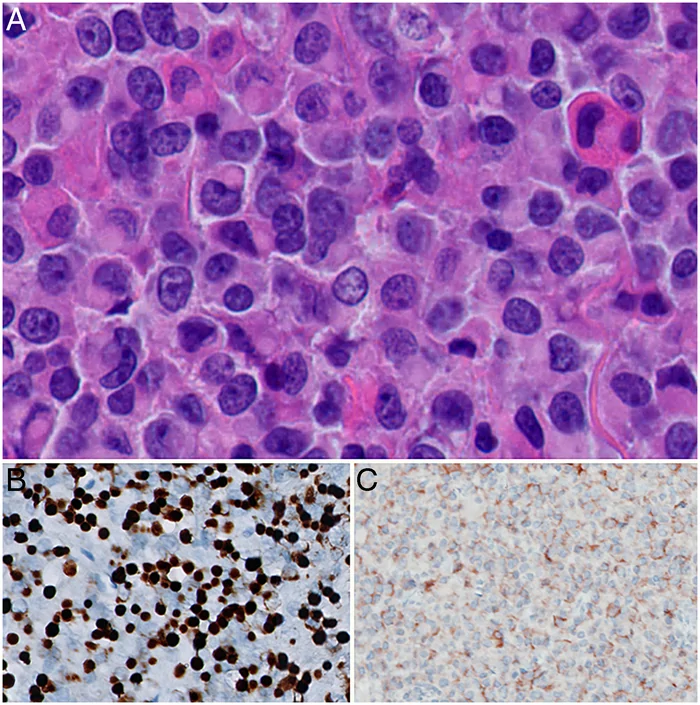
Example of sparsely granulated somatotroph PitNET composed of cells with moderate nuclear pleomorphism and pale eosinophilic cytoplasm. Characteristically, tumor cells show cytoplasmic fibrous bodies that appear as juxtanuclear, pale, globular, eosinophilic structures (A) and are strongly positive for CAM 5.2 (B). Growth hormone immunoreactivity is faint and not observed in all tumor cells (C).

### Χ-Linked Acrogigantism

X-linked acrogigantism (XLAG) is usually observed in females and is characterized by early-onset (below the age of 4 y) pituitary gigantism due to marked GH excess associated, in most cases, with hyperprolactinemia. Other frequently observed clinical features include acral enlargement, coarse facial features, increased appetite and, less frequently, acanthosis nigricans, sleep apnea/snoring and hyperhidrosis.^81,82^ GH and prolactin excess are caused by mixed somatotroph-lactotroph PitNETs, which show a typical sinusoidal and lobular architecture with frequent calcifications and follicle-like structures.^82^ In a minority of patients, GH hypersecretion is due to GH-cell hyperplasia.

XLAG is a very rare disease with fewer than 40 cases reported.^43^ It is caused by a somatic or germline genomic microduplication of the *GPR101* gene (Xq26.3). The *GPR101* gene (Xq26.3) encodes a G protein-coupled orphan receptor, as it is not currently linked to a known ligand.^43^ In humans, immunohistochemical expression of GPR101-encoded protein and/or *GPR101* gene overexpression has been observed in pituitary glands from fetuses and adolescents but not in adult tissues, suggesting an age-dependent expression.^83,84^ Among PitNETs, *GPR101* was found to be overexpressed only in XLAG patients, absent in patients with sporadic somatotroph PitNETs.^83^ The pathogenic mechanisms of XLAG are not completely understood. It is known that GPR101 is coupled with the G protein and its overexpression induces increased production of cAMP, which may favor the development of pituitary hyperplasia and PitNET.^83,85^

## PARATHYROID GLAND

In about 5% to 10% of cases, parathyroid proliferations present as a multiglandular disease in the setting of either genetically determined hereditary tumor syndromes [MEN1, MEN2A, MEN4, and Hyperparathyroidism-Jaw Tumor (HPT-JT) syndrome] or familial nonsyndromic disorders [familial isolated hyperparathyroidism (FIHP), familial hypocalciuric hypercalcemia (FHH), neonatal severe hyperparathyroidism (NS-HPT)] (Table 3), which predispose individuals to parathyroid hyperfunction through different mechanisms (Box 2).^86,87^

**TABLE 3:** Parathyroid Lesions Presenting in the Setting of Either Hereditary Tumor Syndromes or Familial Nonsyndromic Disorders

Disease	Gene	Encoded Protein	Parathyroid Neoplasm
Syndromic tumor syndrome			
MEN1	*MEN1*	MENIN	Multiple adenomas and carcinoma (very rare)
MEN2A	*RET*	RET	Multiple adenomas
MEN4	*CDKN1B*	p27	Single adenoma
MEN5	*MAX*	MAX	Multiple adenomas
HPT-JT	*CDC73*	Parafibromin	Carcinoma, atypical tumor, and adenoma
Nonsyndromic disorders			
FIHP	MEN1 CASR CDC73 GMC2	MENIN CaSR Parafibromin GCMb	Multiple adenomas
FHH1	CASR	CaSR	Functional disorder
FHH2	GNA11	Gα11	Functional disorder
FHH3	AP2S1	Adaptor protein σ-subunit	Functional disorder
NS-HPT	CASR	CaSR	Hyperplasia

FHH indicates familial hypocalciuric hypercalcemia; FIHP, familial isolated hyperparathyroidism; HPT-JT, hyperparathyroidism-jaw tumor syndrome; MEN, multiple endocrine neoplasia; NS-HPT, neonatal severe hyperparathyroidism.

BOX 2 Features of Hereditary Parathyroid Neoplasms

**Table:** 

Syndrome/Disorder	Usual Parathyroid Pattern	Key Pathological/Clinical Clues	Main Gene/Mechanism
MEN1	Multiglandular, asynchronous or metachronous disease; now regarded as pluriglandular adenomas rather than true hyperplasia.	Suspect in multiglandular disease, especially with other MEN1 tumors; morphology alone does not distinguish it from sporadic adenoma.	MEN1
MEN2A	Usually, single or multiple adenomas, mild hyperparathyroidism occurs in a subset of RET-associated disease.	Think of it when PHPT occurs in a patient with medullary thyroid carcinoma or a known MEN2 background.	RET
MEN4	Usually, a single-gland adenoma; PHPT is the leading manifestation.	Consider when a MEN1-like phenotype is present, but MEN1 testing is negative.	CDKN1B/p27
MEN5	Often, multiglandular adenomas or hypercalcemia are related to MAX-associated tumors.	Pathology is usually nonspecific; clinical context is essential.	MAX
HPT-JT	Often, single adenoma or carcinoma; metachronous lesions can occur.	Sheet-like growth, staghorn vessels, cystic change, eosinophilic cells, perinuclear clearing, nuclear enlargement, and multinucleation. Strongly suspect when you see carcinoma features, cystic change, or parafibromin loss. This is the main syndrome with useful morphologic clues. Parafibromin immunohistochemistry is useful.	CDC73
FIHP	Familial PHPT without syndromic features; may be multiglandular or single-gland.	Diagnosis depends on family history and molecular testing, not histology.	Incomplete penetrance of MEN1, CDC73, CASR, or GCM2 variants.
FHH	Not a true parathyroid neoplasm pattern; biochemical disorder with persistent hypercalcemia.	Parathyroid glands may be normal or mildly hyperplastic; morphology is not diagnostic. Suspect when calcium is high, but urine calcium is low, and PTH is inappropriately normal.	CASR, GNA11, AP2S1.
NS-HPT	Severe early-life parathyroid hyperfunction.	No distinctive histology is needed for diagnosis. Consider in neonates/infants with severe hypercalcemia and skeletal demineralization.	Biallelic CASR mutation.

### Multiple Endocrine Neoplasia Type 1 Syndrome

In MEN1 syndrome, primary hyperparathyroidism (PHPT) is the earliest and most common endocrine disorder, affecting >93% of patients with a 75% penetrance at the age of 50 years and >90% at the age of 70, with no apparent gender predilection. In about two-thirds of cases, it is the first clinical manifestation.^88–92^ Patients characteristically present with pluriglandular involvement, asynchronous or metachronous, traditionally considered as parathyroid hyperplasia. However, as these lesions are clonal proliferations,^93^ the latest WHO classification considers them as pluriglandular adenomas.^94,95^ Currently, the term parathyroid hyperplasia is only applied to rare cases of pluriglandular diseases in the context of secondary and tertiary hyperparathyroidism.^95^

Parathyroid adenomas in MEN1 syndrome show the same morphologic features as non-MEN1-associated adenomas, so simple morphologic analysis is not useful for identifying MEN1 patients. Parathyroid adenomas appear as well-circumscribed or encapsulated epithelial proliferations forming sheets, nests, trabeculae, or pseudofollicles associated with a delicate capillary network with sparse or absent fat stroma. A rim of atrophic parathyroid tissue can be observed in more than half of cases. Tumors are usually composed of chief cells, although a mixture of chief, transitional, oncocytic, and clear cells is frequently observed (Fig. 6). Cases showing broad fibrous bands, necrosis, or elevated mitotic counts in absence of invasive growth are defined as atypical parathyroid tumors (Fig. 7). MENIN immunohistochemistry may be useful in the differential diagnosis between MEN1-associated and MEN1-unassociated adenomas, but it is worth noting that loss of MENIN expression can also occur in sporadic cases.^96,97^

**FIGURE 6:**
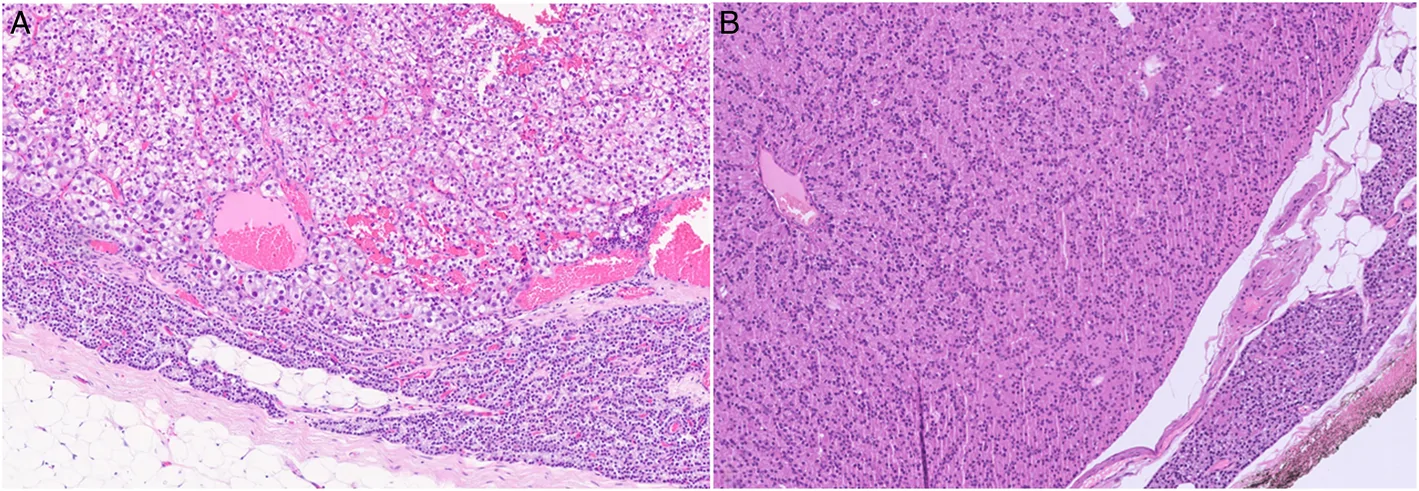
Parathyroid adenomas composed of chief clear cells (A) and oxyphil cells (B). In both cases, a rim of non-neoplastic parathyroid tissue is observed at the periphery.

**FIGURE 7:**
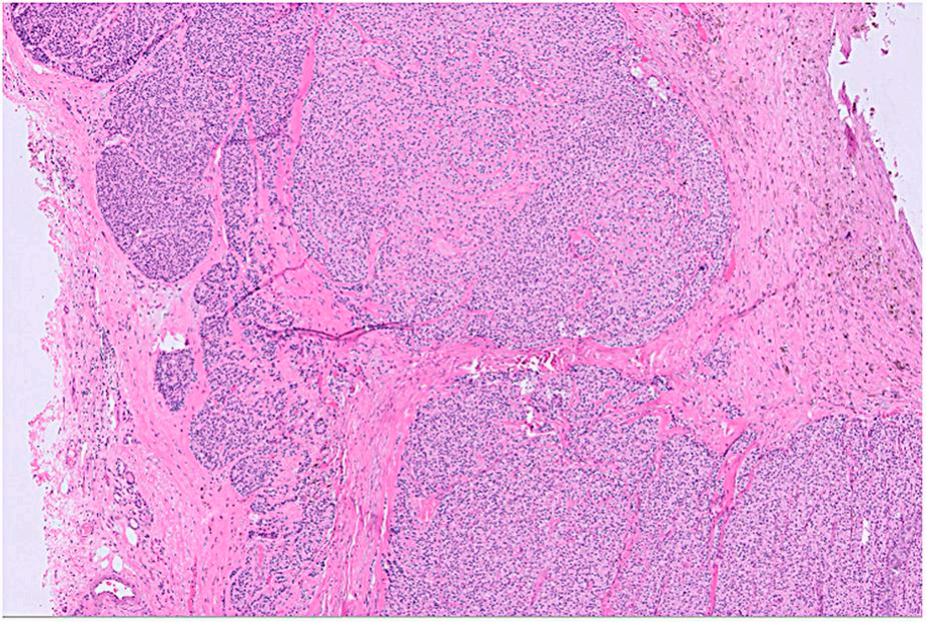
Atypical parathyroid tumor characterized by the presence of fibrous bands separating the parathyroid parenchyma in lobules without unequivocal signs of malignancy, such as capsular, vascular, or perineural invasion or invasion into adjacent structures.

### Multiple Endocrine Neoplasia Type 2 Syndrome

Multiple endocrine neoplasia type 2 (MEN2) syndrome is an autosomal dominant tumor syndrome caused by activating mutations in the *RET* proto-oncogene, located on chromosome 10q11.2,^98^ and includes 2 subtypes: MEN2A (95%) and MEN2B (5%). MEN2A is the classic form and is characterized by the development of medullary thyroid carcinoma (MTC), often associated with adrenal paraganglioma and/or parathyroid tumors. Variants of MEN2A include isolated MTC (formerly known as familial MTC),^99^ MEN2A with cutaneous lichen amyloidosis, and MEN2A with Hirschsprung disease. MEN2B is a rarer subtype characterized by the presence of aggressive MTC associated with adrenal paraganglioma, oral and intestinal ganglioneuromas, and marfanoid habitus. The annual incidence of MEN2A is about 1 case per 1,973,500 people, while that of MEN2B accounts for 1 case per 38,750,000 people. The prevalence of MEN2 is estimated at about 1 per 30,000 people. The M:F ratio is about 1:1, although some studies showed a slight female predominance.^99,100^ Patients with MEN2 present with signs and symptoms of MTC. Hyperparathyroidism is observed in 20% to 30% of patients with MEN2A, except for patients with isolated familial MTC (a subtype of MEN2A), but not in patients with MEN2B.^101^

Hyperparathyroidism usually presents at the average age of 38 years, several years after the diagnosis of MTC, which is the earliest and most common manifestation of the syndrome. Hyperparathyroidism is typically mild and may be due to either single or multiple parathyroid adenomas.^102,103^ These parathyroid tumors show the same morphologic features of sporadic and MEN1-related neoplasms.

At the genetic level, MEN2A and MEN2B are caused by distinct patterns of RET amino acid substitutions. The *RET* gene is a proto-oncogene that encodes a tyrosine kinase receptor related to cellular proliferation, differentiation, and survival.^104^ *RET* mutations in MEN2A syndrome affect extracellular cysteine residues, promoting ligand-independent dimerization and constitutive RET activity, while in MEN2B, *RET* mutations, caused by a single amino acid change in the tyrosine kinase domain, release RET autoinhibition, producing a more active RET kinase activity that can promote signaling in the absence of a ligand.^98^ In addition to producing intrinsic biochemical changes in the RET structure and activation, these *RET* mutations also alter RET cellular location, interactions, and mechanisms of downregulation that can prolong or mislocate RET activity. As a result, MEN2 syndrome is not a unique disorder but includes different subtypes that differ in tumor aggressiveness and in the association of tumors with clinical features.

### Multiple Endocrine Neoplasia Type 4 Syndrome

Primary hyperparathyroidism is the leading symptom of MEN4 syndrome, being observed in 80% to 90% of patients. Compared with MEN1, patients are more frequently females and at a more advanced age (mean age of 60 y) if compared with MEN1 patients.^105,106^ In most cases, hyperparathyroidism is due to uniglandular adenoma, and no relapse after parathyroidectomy has been observed.^24^ Parathyroid adenomas are not morphologically different from sporadic cases.

### Multiple Endocrine Neoplasia Type 5 Syndrome

Hypercalcemia is frequently observed in patients with MEN5 syndrome and, in most cases, it is related to multiglandular parathyroid adenomas, which show the same morphologic features of sporadic counterparts.^30^ Occasionally, hyperparathyroidism has been described in patients with ectopic secretion of PTH or PTHrP by other MAX-associated tumors.^107^

### Hyperparathyroidism-Jaw Tumor Syndrome

Hyperparathyroidism-jaw tumor (HPT-JT) syndrome is an autosomal dominant disorder caused by pathogenic germline mutations or deletions in the *CDC73* gene, leading to parathyroid adenoma or carcinoma development in 95% of cases, fibro-osseous jaw lesions in 25% to 50% of patients, and, more rarely, uterine and renal tumors, including hamartomas and Wilm tumor.^108,109^ HPT-JT syndrome is a rare cause of hyperparathyroidism, accounting for about 0.2% of unselected parathyroid tumors.^110^ However, primary hyperparathyroidism is the usual presentation of HPT-JT (85% of patients) at a median age of 26 years.^111^ In the majority of cases (60% to 80%), patients present a single tumor, either adenoma or carcinoma, but metachronous tumors can frequently appear during follow-up.^112–114^

*CDC73* is located on chromosome 1q31.2 and encodes the nuclear protein parafibromin, a critical component of the polymerase-associated factor 1 complex (PAF1C) that binds to RNA polymerase II and regulates transcription.^109–116^ Protein-truncating variants of *CDC73*, seen in up to 80% of germline mutations in HPT-JT,^117^ produce altered parafibromin, which anomalously interacts with nuclear beta-catenin and increases the expression of cyclin D1 that in turn stimulates proliferation.^118,119^

Parathyroid tumors observed in patients with HPT-JT syndrome show a sheet-like growth pattern, associated with peculiar vasculature (staghorn vessels) and cystic changes, and are composed of cells with eosinophilic cytoplasm, perinuclear cytoplasmic clearing, and nuclear enlargement with occasional multinucleation (Fig. 8).^110,112^ In 31% of cases, tumors show the diagnostic features of parathyroid carcinoma, including unequivocal local invasion, vascular space invasion, and/or metastasis.^95^ The observation of the above-described morphologic features strongly suggests performing parafibromin immunohistochemistry, which shows parafibromin loss reflecting biallelic *CDC73* mutations. It is worth noting that tumors are considered parafibromin-deficient when parafibromin nuclear staining in neoplastic cells is completely lost, despite parafibromin nuclear expression in non-neoplastic cells.^110,120,121^ Since parathyroid carcinoma is extremely rare outside the setting of germline or somatic *CDC73* mutation, the possibility of HPT-JT syndrome should be considered in all patients with parathyroid carcinoma, even in the absence of a known family history.^110,120^ However, it is important to recall that some *CDC73* point mutations can be associated with preserved parafibromin immunoreactivity. Consequently, positive staining for parafibromin in parathyroid neoplasms does not exclude, per se, the diagnosis of HPT-JT syndrome. In these cases, PGP9.5 immunohistochemistry may be helpful, as diffuse, strong expression is frequently observed and increases the sensitivity of the parafibromin test.^114,120,122^

**FIGURE 8:**
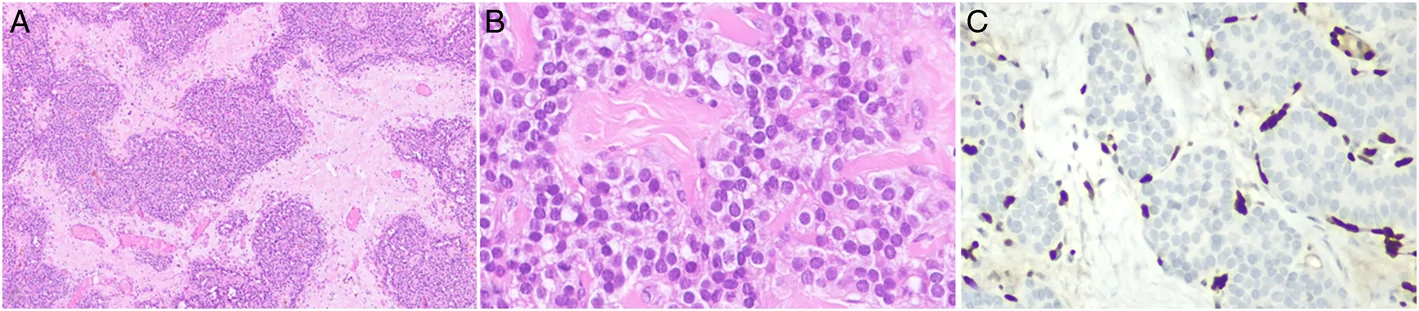
Parafibromin-deficient parathyroid tumor shows characteristic features, including fibrosis and evident vasculature (A) and granular eosinophilic cytoplasm and perinuclear halo (B). Parafibromin immunostain is globally lost in tumor cells, whereas it is retained in endothelia and stromal cells (C).

In summary, young age (especially <40 y) of onset, large tumors, cystic change, parathyroid carcinoma features, markedly elevated serum parathormone levels, and severe hypercalcemia should suggest the possibility of HPT-JT syndrome even when the family history is silent. Parafibromin negativity strongly supports the diagnosis of HPT-JT syndrome and the indication for genetic testing.^110,123^ As a final consideration, it is also important to recall that parafibromin loss can also be observed in patients with *CDC73* somatic mutations. Therefore, it should not be considered a definitive marker of HPT-JT syndrome without genetic confirmation.

Ossifying fibromas and other clinical manifestations show low incidence and generally develop after parathyroid tumor onset. For this reason, the pathologist's challenge mainly resides in the identification of this tumor syndrome when approaching the parathyroid lesion.

### Familial Nonsyndromic Disorders Causing Primary Hyperparathyroidism

These conditions include 3 different diseases, in which parathyroid neoplasms are not associated with other nonparathyroid disorders. The identification of these syndromes requires knowledge of the clinical context and mutation analysis.

- Familial isolated hyperparathyroidism (FIHP) is a nonsyndromic cause of primary hyperparathyroidism resulting from incomplete penetrance of pathogenic variants in *MEN1*, *CDC73*, or *CASR* genes.^87^ In addition, activating mutations in *GCM2*, which is located on chromosome 6p24.2, have been reported in about 20% of patients with FIHP.^124^
- Familial hypocalciuric hypercalcemia (FHH) is a heterogeneous disorder with 3 variants caused by inactivating mutations of 1 of 3 genes involved in calcium-sensing machinery:^125,126^
*CASR* encoding calcium-sensing receptor (FHH1, or type 1);
*GNA11* is involved in calcium-sensing receptor signaling (FHH2, or type 2);
*AP2S1* regulates calcium-sensing receptor endocytosis (FHH3, or type 3).
  Lifelong elevations in serum calcium concentrations, with low urinary calcium excretion and normal circulating parathyroid hormone levels, are present in 80% of affected individuals. Patients are generally asymptomatic, although pancreatitis or chondrocalcinosis may develop in some affected adults.
- Neonatal severe hyperparathyroidism (NS-HPT) is caused by either homozygous or compound heterozygous *CASR* mutations. Symptoms usually manifest within the first 6 months of life, and patients show severe hypercalcemia, skeletal demineralization, bony deformities, fractures, constipation, and dehydration.^87^

## THORACIC NEUROENDOCRINE TUMORS

The vast majority of lung and thymic neuroendocrine tumors (NETs) are sporadic, but in a minority of patients, they can arise in the context of MEN1 syndrome, and rare cases have been described in patients with MEN4 or Cowden syndrome.^127^ Since there are no specific morphologic features characterizing both lung and thymic NETs as belonging to a hereditary tumor syndrome, we will discuss the main clinico-pathologic features of these neoplasms grouped by organ and not by syndrome.

### Lung Neuroendocrine Tumors

In MEN1 patients, lung NETs are more frequent than previously thought, being observed in 4.8% to 6.6% of cases, with an estimated prevalence of 2% to 8%. The median age at diagnosis is 43 years, and no relation with smoking has been demonstrated.^128–130^ In most cases, lung NETs are clinically asymptomatic and identified radiologically during screening. However, rare cases present with endocrine symptoms related to hormonal hypersecretion, including carcinoid syndrome, Cushing syndrome, and acromegaly.^131^

Both sporadic and MEN1-related lung NETs are classified using the same WHO criteria, including mitotic count and the presence of necrosis. However, we are convinced that the Ki67 proliferative index should always be included in the pathology report. Using this approach, most MEN1-related lung NETs are classified as typical carcinoid (TC)/NET G1,^130^ which are generally associated with indolent behavior and good prognosis,^132^ with a reported 10-year survival of ∼88%.^129^ In one series, the presence of lung NET did not seem to decrease patients’ overall survival.^129^ However, it is worth noting that distant metastases have been observed in 3% to 16% of patients.^128,129^ Prognostic factors worsening survival seem to include atypical carcinoid (AC) subtype and the presence of lymph node and/or distant metastases.^129^ Precursor lesions such as tumorlets and bronchopulmonary neuroendocrine cell hyperplasia (Fig. 9), as well as multifocal tumor occurrence, were noted in 3 tumors in one article.^130^

**FIGURE 9:**
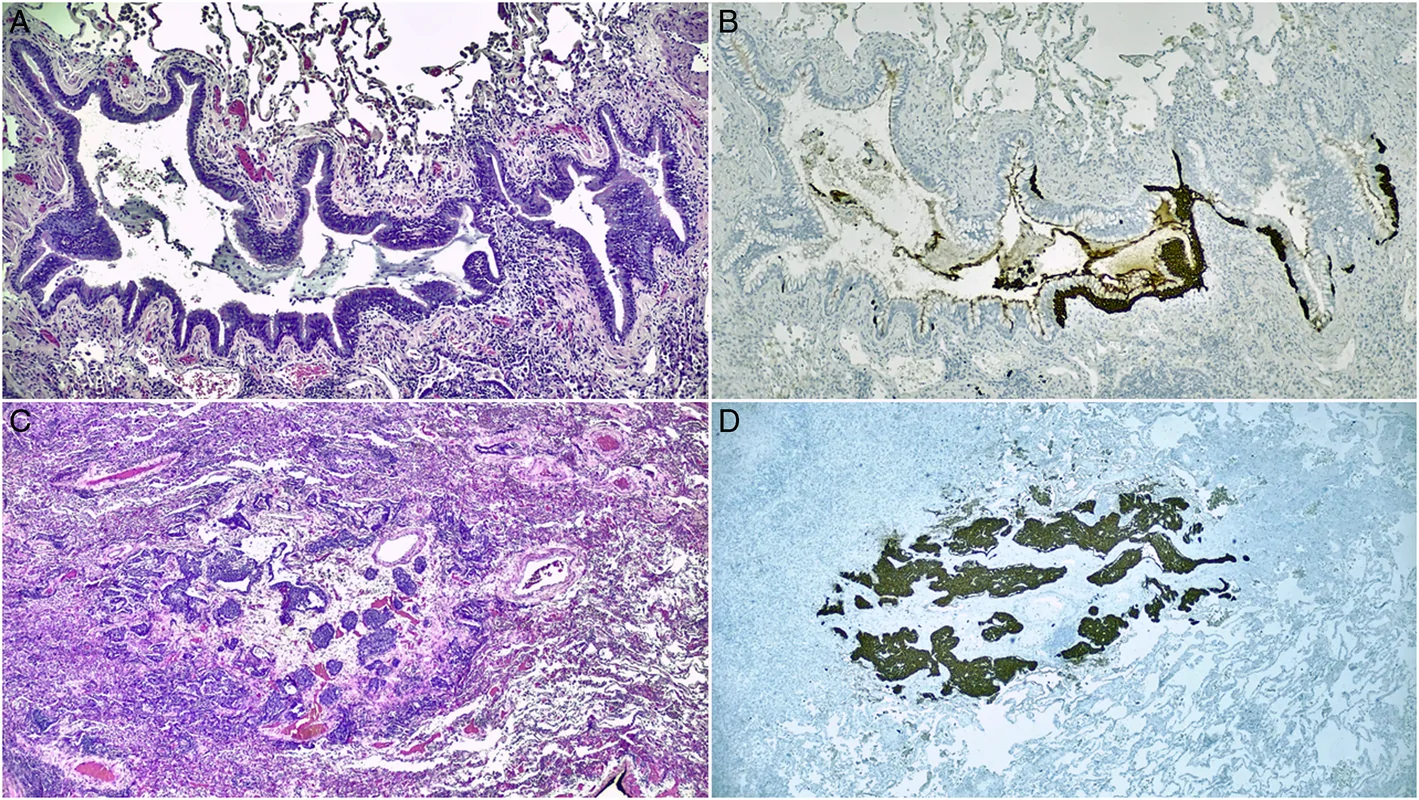
Precursor neuroendocrine lesions in a patient affected by MEN1 syndrome include bronchopulmonary neuroendocrine cell hyperplasia characterized by a proliferation of neuroendocrine cells within the bronchiolar mucosa (A), strongly positive for chromogranin A (B), and by tumorlets, which are nodular proliferations of neuroendocrine cells invading beyond the bronchiolar wall but less than 5 mm in size (C) and positive for chromogranin A (D).

In addition to MEN1 syndrome, lung NETs may also arise in Cowden syndrome (CS). Indeed, a patient with confirmed CS developing multiple bronchial TCs and tumorlets has recently been described.^133^ CS is a rare autosomal dominant inherited condition caused by mutations in the *PTEN* tumor suppressor gene located on 10q22-23.^134^ Patients with CS have an increased risk of developing several different benign and malignant tumors, including follicular thyroid, breast, endometrial, and renal cell neoplasms. In addition, they can also show macrocephaly, mental retardation, lipomas, trichilemmomas, fibromas, papillomatous skin lesions, and hamartomatous polyps of the gastrointestinal tract.^134,135^

### Thymic Neuroendocrine Tumors

The estimated prevalence of thymic NETs in MEN1 patients is 2% to 8%, but they significantly contribute to the morbidity and mortality of MEN1 patients, being aggressive tumors with metastases in more than 50% of cases and responsible for up to 20% of the MEN1-related deaths.^131^ For this reason, prophylactic thymectomy in young people bearing *MEN1* mutations, with or without signs of hyperparathyroidism, has been proposed with the aim of reducing the risk of thymic NET development and improving prognosis.^131,132^ In any case, patients with MEN1 syndrome should be prudentially screened for thymic NETs by thoracic imaging every 1 to 2 years.^17^ Thymic NETs more frequently affect males in the fifth decade of life^136–138^ and are classified using the current criteria proposed by the WHO that overlaps those of lung NETs.

In addition to MEN1 syndrome, thymic NETs have also been described in the context of *MEN4* syndrome.^23^

## DIGESTIVE SYSTEM

The digestive system is the most common site of origin for neuroendocrine tumors (NETs). This chapter addresses the most suggestive pathologic features of an inherited pathogenesis of digestive NETs. Distinctive morphologic characteristics associated with germline mutations are discussed, and an overview of the diagnostic, prognostic, and predictive significance of immunohistochemical analyses, when useful, is provided. Boxes 3 and 4 summarize morphologic clues suggestive of hereditary NET and hereditary NET patterns in the digestive system.

BOX 3 Morphologic Clues Suggestive of Hereditary NET in the Digestive System

**Table:** 

Site	Morphologic Clue	Why It Suggests Hereditary Disease
Pancreas	Multifocal NETs or numerous microscopic tumorlets.	Hereditary NETs, especially MEN1-associated PanNETs, are often multiple rather than solitary.
Pancreas	Hyperplastic or dysplastic islets in peritumoral tissue.	These are among the strongest histologic clues to a possible MEN1/MEN4 syndrome
Pancreas	Microtumors adjacent to the main lesion.	Supports a field defect/precursor-lesion pattern seen in hereditary cases.
Pancreas	Islets with aberrant insulin and glucagon distribution.	This abnormal endocrine architecture is characteristic of hereditary NETs.
Pancreas/duodenum	Multiple synchronous primaries.	Multiple lesions within the gland or duodenum favor an inherited predisposition.
Small bowel	Several synchronous ileal/jejunal NETs.	Familial small-intestinal NETs are more often multiple and have an earlier onset (but sporadic multiple ileal NETs are not uncommon)
Any well-differentiated NET	Earlier-than-expected presentation and/or multifocality.	Not purely morphologic, but in pathology practice, this combination should prompt consideration of hereditary disease.

BOX 4 Hereditary NET Patterns in the Digestive System

**Table:** 

Syndrome	Most Suggestive Pathology Pattern	Typical Sites/Tumor Types	Key Microscopic Clue(s)
MEN1	Multifocal microtumors, often with a background of multiple microscopic lesions.	Pancreas and duodenum; multiple pancreatic NETs, duodenal gastrinomas, sometimes thymic NETs.	Small synchronous lesions, neuroendocrine cell hyperplasia, and heterogeneous peptide expression; the overall pattern is more important than a single diagnostic morphology.
VHL	Pancreatic NETs with clear-cell change, often with a background of cystic/serous lesions.	Pancreas; usually nonfunctioning PanNETs, sometimes mixed serous cystadenoma-PanNET lesions.	Clear-cell cytology is the strongest morphologic clue; tumors are often solid, trabecular, or glandular, and may be multiple.​​​​​​Immunohistochemistry for CAIX may be of use.
NF1	Duodenal somatostatinoma pattern, often periampullary.	Duodenum/periampullary region; somatostatin-producing NETs predominate.	Psammoma bodies are a classic clue, especially in duodenal somatostatinomas; otherwise, morphology is usually not distinctive.
TSC	Pancreatic NETs are sometimes small and multifocal, but usually without a unique signature.	Pancreas; rare pNETs, sometimes insulinoma or gastrinoma.	No highly specific morphology in most cases; suspicion rises when PanNETs occur in a young patient or as multiple lesions, especially when accompanied by other syndromic lesions.

### Pancreas

The reported incidence of inherited pancreatic neuroendocrine neoplasms (PanNENs) among the total PanNENs is 10%.^139^ However, recent studies suggest that the actual frequency of germline mutations in these neoplasms may be between 20% and 30%, approaching that of paragangliomas and justifying the introduction of universal germline testing on PanNENs.^140,141^ Early recognition of inherited PanNEN has significant clinical value, both for managing at-risk family members and for guiding therapeutic strategies. Indeed, new molecularly targeted options are available in these patients.^141^

Pathologists play a central role not only in the accurate diagnosis and subtyping of PanNENs but also in recognizing specific morphologic features that may point to a germline etiology. In addition, pathologists are essential in selecting the most appropriate histologic material for subsequent molecular analyses.

Most of the available data regarding germline mutations in PanNENs concern neuroendocrine tumors (PanNETs). However, germline mutations have also been identified in ∼4.5% of pancreatic neuroendocrine carcinomas (PanNECs). These mutations frequently involve key cancer genes such as *TP53*, *RB1*, and those implicated in homologous recombination repair (HRR), which aligns with the established overlap in pathogenesis between PanNECs and non-neuroendocrine pancreatic carcinomas.^3,142,143^

#### Hereditary Syndromes Associated With PanNET

The most common germline mutations in PanNET are typically observed in familial syndromes. The most frequent hereditary syndromes are listed below in order of their frequency among PanNET cases. However, it should be noted that germline mutations in DNA repair genes (eg, *BRCA2*, *MUTYH*, *CHECK2*, and others) have been found in a non-negligible proportion of pancreatic tumors.^139^ In addition, PanNETs have been reported, albeit less frequently, in association with Lynch syndrome^144,145^ and, in a single report, in association with SDH deficiency.^146^

##### Multiple Endocrine Neoplasia Type 1 Syndrome

The genetic and clinico-pathologic features of MEN1 syndrome have been described in the previous chapters. Most MEN1-related PanNETs are nonfunctioning; among functioning tumors, insulinomas are the most common. MEN1-related gastrinomas typically arise in the duodenum, and their occurrence in the pancreas is very rare.

##### Multiple Endocrine Neoplasia Type 4 Syndrome

As mentioned above, MEN4 is associated with germline mutations in *CDKN1B* and is characterized by a clinical phenotype similar to that of MEN1. Hyperparathyroidism is the most frequent manifestation, followed by anterior pituitary tumors. MEN4-related PanNETs have rarely been reported, and thyroid and adrenal involvement is even rarer.^147^

##### Von Hippel-Lindau Syndrome

VHL syndrome is due to the germinal mutations of the *VHL* gene, located on chromosome 3p25-26 and encoding a protein (pVHL). pVHL is a key regulator of the hypoxia-inducible factors (HIF family), ensuring the ubiquitination and, ultimately, the degradation of HIF-α in conditions of normoxia.^148^ In addition, this protein interacts with fibronectin-mediated extracellular matrix assembly and seems to be involved in p53 regulation.^148^ The clinical manifestations of this syndrome depend, also in terms of severity, on specific mutation of the *VHL* gene,^149^ and include: cerebral, spinal, and retinal hemangioblastomas, clear-cell renal carcinomas, pheochromocytomas, PanNETs, endolymphatic sac tumors, and cystic lesions in various organs.^148^

##### Neurofibromatosis Type 1

NF1 is related to a germline mutation in the NF1 gene, located on chromosome 17q11.2 and encoding neurofibromin. Neurofibromin is ubiquitously expressed, with the highest levels in the central nervous system. Neurofibromin interacts with intracellular pathways regulating cell proliferation (RAS/MAPK and PI3K/AKT/mTOR). The syndrome exhibits a highly variable phenotype, depending on the specific molecular alteration. Most clinical manifestations of NF1 involve the nervous system and skin, but it is, in fact, a multisystem disease.^150^ Among NF1-related tumors, the most frequent are gliomas (of the brainstem and of the optic pathway), neurofibromas, and malignant nerve sheath tumors. NETs have been reported to arise in the context of NF1 syndrome, with a prevalence of duodenal tumors (D-cell, somatostatin-secreting NETs of the ampullary region) over PanNET.^151–153^ A few case reports and case series of PanNET have been reported.^154,155^

##### Glucagon Cell Hyperplasia and Neoplasia

GCHN is a recently recognized extremely rare syndrome related to the inactivating germline mutation of *GCGR* gene, located on chromosome 17q25.3, which encodes for the glucagon receptor.^156^ The disease is transmitted in an autosomal recessive pattern, and patients with biallelic mutations present with upper abdominal discomfort due to pancreatic enlargement from GCHN. Hyperglucagonemia is present, without symptoms of the glucagonoma syndrome.^156,157^ The glucagon receptor is mainly expressed by hepatocytes. Glucagon signaling plays a crucial role in glucose, amino acid, and lipid metabolism. Biallelic inactivation of the *GCGR* gene leads to hyperaminoacidemia and nonfunctional hyperglucagonemia. The absence of a functional glucagon receptor in pancreatic alpha cells is associated with a pancreatic disorder called GCHN (Mavash disease).^158,159^ The pathogenetic link between glucagon hypersecretion and alpha-cell hyperplasia and the transition to glucagon-producing NETs in the pancreas remains controversial. Recently, however, it was shown in a case with GCHN that a glucagon-positive NET had developed a somatic mutation of the *MEN1* gene,^160^ genetic process known for its neoplastic potential in PanNET. The development of metastasis to the liver has been recently described in 1 case.^160^

##### Tuberous Sclerosis Complex

Tuberous sclerosis complex (TSC) is a genetically determined disease caused by germline mutations in the *TSC1* (located on chromosome 9q34) or *TSC2* (located on chromosome 16p13) genes, which encode hamartin and tuberin, respectively. It is a rare multisystem disease, transmitted with an autosomal dominant pattern and characterized by the presence of benign tumors or hamartomas, skin abnormalities, and neurological manifestations. The most involved organs are the brain, heart, lungs, skin, kidneys, and eyes.^161^ PanNETs are rare findings in patients with TSC and are usually incidental during follow-up of the disease, which includes abdominal MRI every 1 to 3 years to assess for renal lesions (angiomyolipomas and renal cysts).^162^ The frequency of TSC-associated PanNETs has been reported to vary between 0.65% to 9%, and, in a quaternary pediatric center, the prevalence of PanNETs in children affected by TSC was 11 out of 267 patients (4.1%).^163^ There is ongoing debate on whether the association is just related to an increased diagnostic sensitivity due to the periodical MRI or there may be a causal relationship between TSC and PanNETs.^164^ TSC-associated PanNETs may arise in the presence of both *TSC1* and *TSC2* mutations and are usually nonfunctioning, although 10 cases of TSC-associated insulinomas have been described.^165^ At present, no systematic studies are available detailing the histopathologic features of TSC-associated PanNETs, and no established guidelines for their treatment have been released.

In addition to these conditions, familial insulinomatosis, related to genetic defects of V-Maf avian musculoaponeurotic fibrosarcoma oncogene homolog A (MAFA), may cause very rarely multiple insulinomas.^166–169^ A case of PanNET and a mixed acinar cell carcinoma/PanNET were described in the context of Cowden syndrome,^170,171^ while a PanNET with loss of MAX nuclear staining reflecting copy number neutral loss of heterozygosity of *MAX* gene has recently been described.^172^

#### Pathologic Features of Inherited PanNETs

In daily diagnostic practice, there are a few hints that pathologists should be able to recognize, suspect, or confirm the inherited nature of a PanNET. Such careful practice should be integrated into the multidisciplinary management of patients to achieve the most comprehensive understanding of the disease. It should nevertheless be recognized that sporadic PanNETs and tumors associated with inherited syndromes do not differ in terms of disease-specific patients’ outcome.^173,174^

- Multiplicity: the presence of multiple tumors is a well-established feature of MEN1-associated PanNETs. Around 85% of PanNETs in MEN1 patients are multiple (Fig. 10), with a mean of 3.2 tumors per patient.^173,175^ In another study, the number of microtumors in 5 distal pancreatectomy specimens varied between 26 and 39.^176^ The same has been demonstrated for PanNETs associated with VHL (Fig. 11).^177,178^ In general, the presence of multiple PanNETs, especially in young patients, should prompt a complete clinical assessment and consideration for genetic counseling.^179^ Furthermore, PanNETs arising in hereditary syndromes, and above all in MEN1, can show cystic appearance or cystic changes/degeneration.^180,181^
- Specific features of neoplastic cells: histologically, the morphology of VHL-associated PanNET may be highly suggestive, as neoplastic cells often exhibit a clear, abundant, microvacuolated cytoplasm, giving the neoplasm a “clear-cell” appearance (Fig. 11).^182^ Interestingly, this is associated with overexpression of carbonic anhydrase IX (CAIX), a protein involved in the HIF1 pathway, which is dysregulated in VHL syndrome.^183^ Indeed, these aspects may mimic a metastatic clear-cell carcinoma of the kidney. Conversely, the possibility of synchronous renal cell carcinoma and PanNET in the context of VHL syndrome should not be overlooked. To distinguish the 2 entities, immunostains for general neuroendocrine markers are mandatory. The immunohistochemical panel may also include a kidney-specific marker, but keep in mind that PAX8 may be expressed in PanNET when using polyclonal antibodies.^184^ To be noted, a clear-cell-like lipid-rich variant of PanNET has been described even in MEN1 patients;^185^ in such cases, the expression of alpha-inhibin, which is exclusive of VHL-related PanNET, may be used as a helpful tool for differentiating the 2 conditions.^186^
- Hormone expression patterns: the most frequently identified hormone of MEN1-related PanNETs is glucagon, followed by PP, insulin, and somatostatin (Fig. 12).^179^ Multihormonality is also frequent. Ectopic hormone production, such as gastrin, calcitonin, and ACTH, may rarely occur.^158,179,187,188^
- Features of non-neoplastic islets: in MEN1 and VHL, non-neoplastic islets are usually normal.^189^ In GCHN, many islets are enlarged, showing alpha-cell hyperplasia with frequent imperceptible transformation to microtumors and ultimately to glucagon-positive macrotumors (Fig. 13).^179,190,191^

**FIGURE 10:**
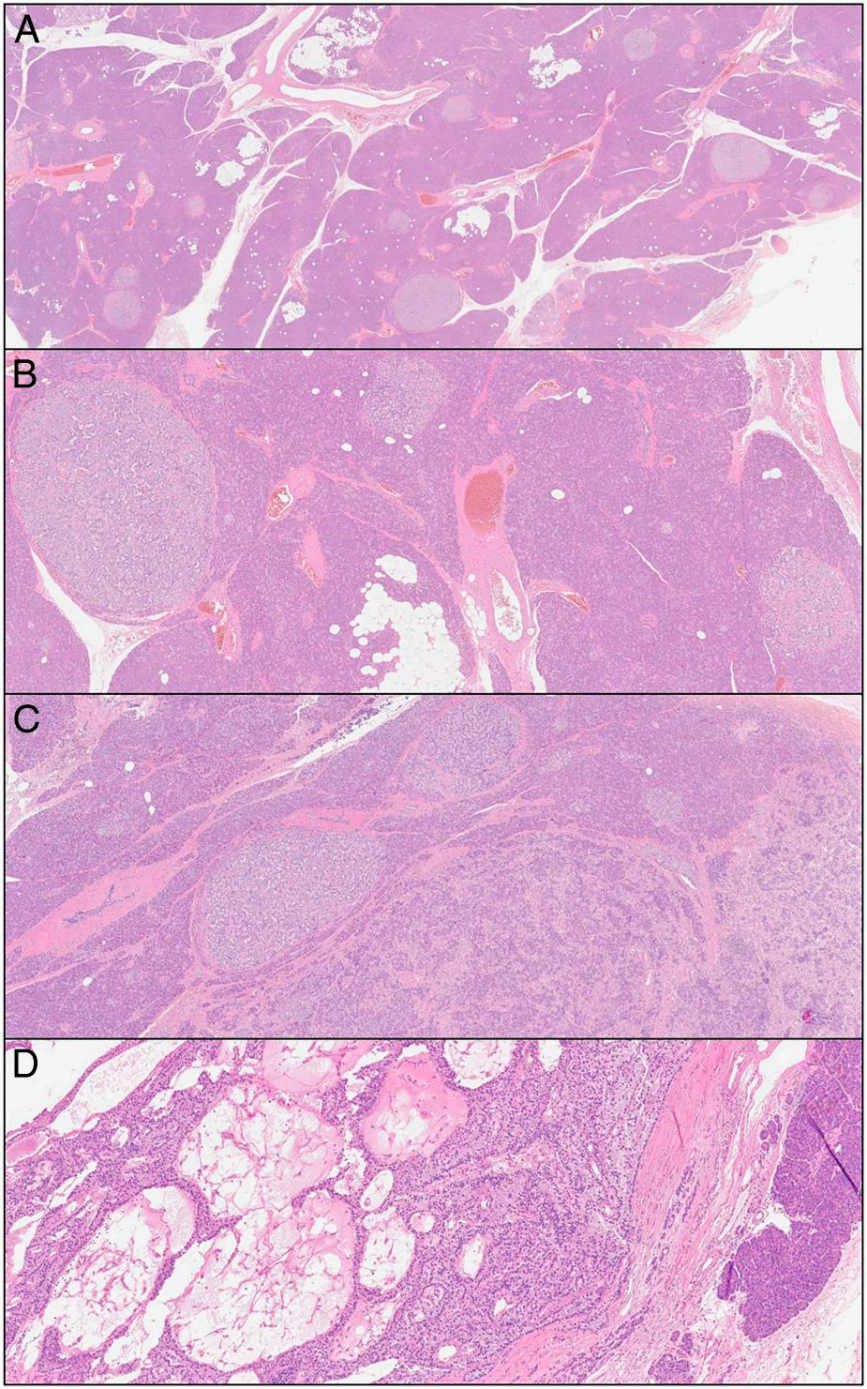
In patients affected by MEN1 syndrome, multiple PanNETs are typically found. They can range from a few millimeters (A and B) to larger, macroscopically visible PanNETs (C). Sometimes, PanNETs can show cystic aspects/degeneration (D).

**FIGURE 11:**
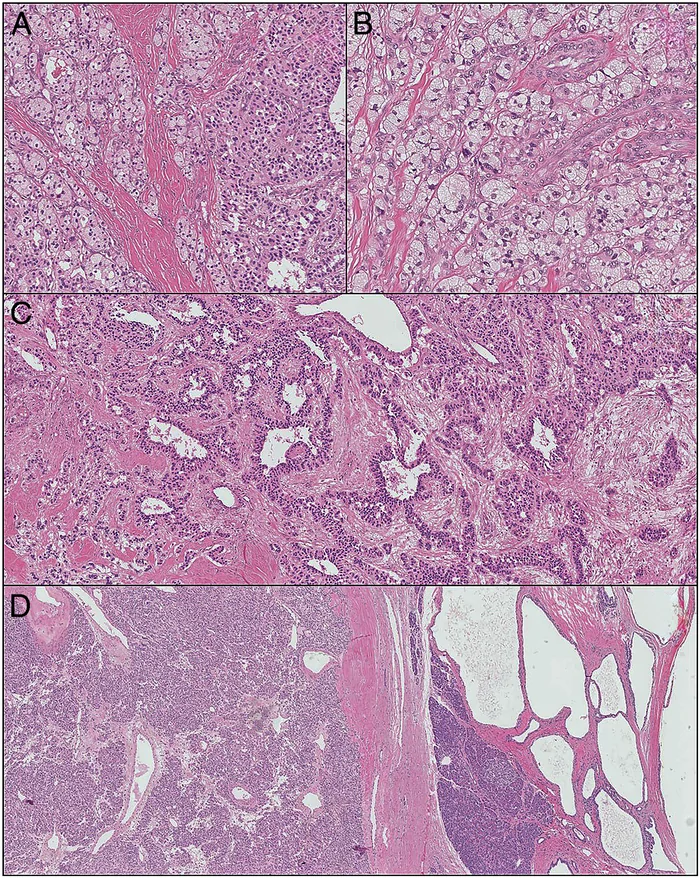
This figure summarizes some of the most typical aspects of pancreatic neoplasms in patients with VHL syndrome. Co-existence of conventional and clear-cell morphology (A), pure clear-cell morphology (B), and pseudo-glandular architecture (C). In addition, the co-existence of PanNET and serous cystic neoplasm is a typical association found in the pancreas of patients with VHL syndrome (D).

**FIGURE 12:**
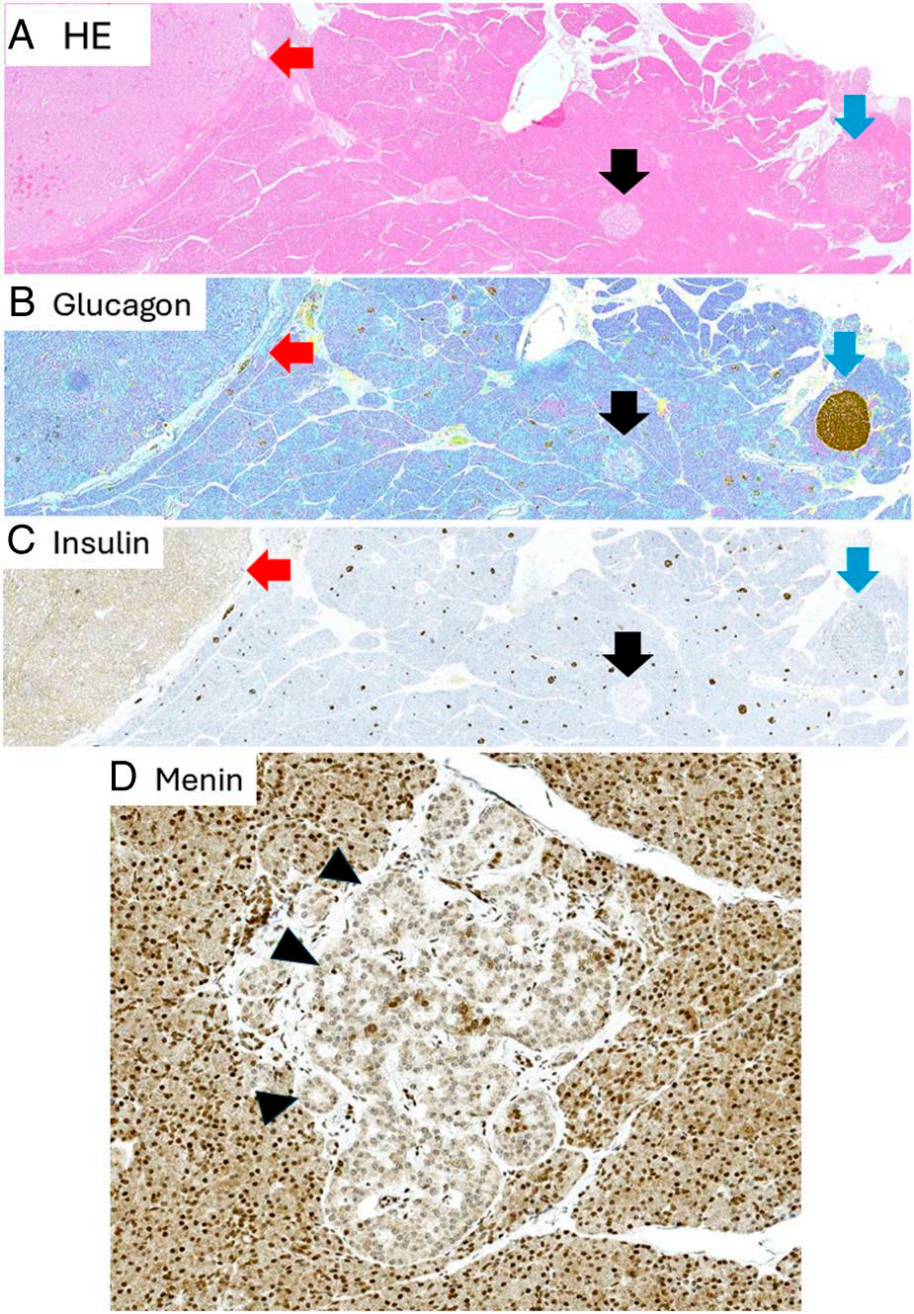
Margin of a pancreatic macro-PanNET in a patient affected by MEN1 syndrome (A–C, red arrows), which is negative for glucagon (B) and immunoreactive for insulin (C). Two other micro-PanNETs can be observed (A–C, black and blue arrows). One microtumor stains (blue arrows) for glucagon (B), the other microtumor shows no hormone positivity (black arrows). The nuclear loss of MENIN positivity in the microtumor indicates a MEN1 gene mutation (black arrow heads) (D).

**FIGURE 13:**
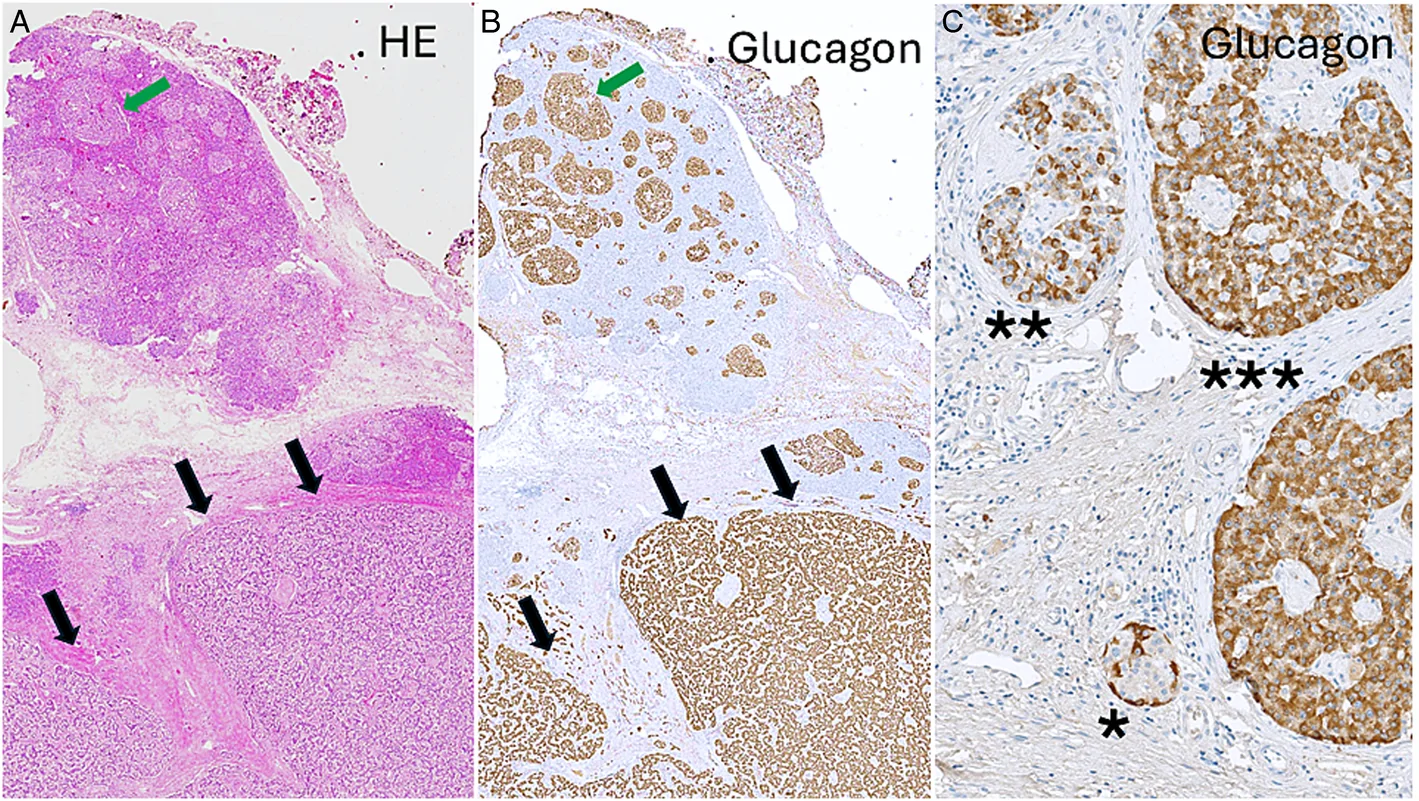
Glucagon cell hyperplasia neoplasia (GCHN, Mavash disease). A, Low power view shows pancreatic tissue with small and large islets and microtumors (upper half, green arrows) and the margin of a macrotumor (lower half, black arrows). Immunostaining for glucagon (B) shows positivity in the macro-PanNET tissue, micro-PanNETs, and islets. C, Glucagon staining shows transition of islet with normal peripheral glucagon cell distribution (*) to islet with glucagon cell hyperplasia (**) to glucagon cell microtumor (***).

##### The Role of Immunohistochemistry in the Diagnosis of Inherited PanNET

Besides its central role in assessing diagnosis, prognosis, and prediction of response to therapy in NENs,^184^ immunohistochemistry may be of significant help, although evaluating multiple stains is notoriously difficult due to uncertain specificity. For instance, abnormal nuclear positivity for menin in the context of multiple PanNETs has been described in so-called preneoplastic lesions in pancreatic islets, suggesting a MEN1-related disease.^192,193^ Altered menin expression is observed in MEN1-associated PanNETs (Fig. 12), but it is also frequently seen in sporadic PanNETs.^194^ In a similar morphologic setting, loss of p27 nuclear immunostaining suggests MEN4.^23^ Moreover, as mentioned before, overexpression of proteins involved in the HIF pathway, such as CAIX and alpha-inhibin, can be a clue to a VHL-related tumor.^183,186^ Loss of tuberin expression has been reported in a PanNET related to TSC,^195^ and tuberin immunohistochemistry has been recently validated as a surrogate for genetic analysis.^196^ Finally, SDHB and mismatch repair (MMR) protein immunohistochemistry may unveil an inherited PanNET in the context of inherited SDH deficiency or Lynch syndrome, respectively.^144–146^

### Stomach

Most gastric NETs are composed of histamine-producing enterochromaffin-like (ECL) cells and are classified into 5 subtypes based on the clinicopathologic background in which they arise. This is defined by the morphology of peritumoral oxyntic mucosa, the presence of antral G-cell hyperplasia, hypergastrinemia, the setting of MEN1 syndrome, hypo- or achlorhydria, autoantibodies directed against parietal cells and/or intrinsic factor, and megaloblastic anemia.^197–199^ Among ECL-cell NETs, type 2 specifically arises in the context of MEN1 syndrome and currently accounts for about 1% of all ECL-cell NETs.^200^ Patients with type 2 ECL-cell NET present with hypergastrinemia due to a duodenal or, more rarely, pancreatic gastrinoma. It is important to note that patients with sporadic gastrinoma do not develop gastric NETs, suggesting that the genetic changes present in MEN1 patients are essential for predisposing ECL cells to gastrin-mediated proliferation.^201^

Tumors are usually multiple, less than 2 cm in size, mainly confined to the mucosa and submucosa, and of grade 1 or grade 2.^202–204^ As other ECL-cell NET types, tumors show microlobular and/or trabecular pattern of growth and are composed of uniform cells with monomorphic round nuclei showing the typical “salt and pepper” chromatin distribution, small nucleoli, and abundant granular eosinophilic cytoplasm. Necrosis is absent, and mitoses are rare (Fig. 14).^202^ Under gastrin stimulus, peritumoral oxyntic mucosa becomes thicker (hypertrophic hypersecretory gastropathy) and different types of hyperplastic and dysplastic ECL-cell proliferations are observed in about 85% and 20% of cases, respectively.^202,205^ Since the morphology of ECL-cell NETs overlaps that of other ECL-cell NETs, the evaluation of peritumoral mucosa associated with information on gastrin serum level is mandatory for the correct diagnosis.^199^ In patients with sporadic gastrinoma, hyperplastic ECL-cell proliferations can also be observed, while dysplastic lesions are extremely rare (2%) and ECL-cell NETs do not apparently develop underlying, once again, the relevance of genetic background in the pathogenesis of gastrin-related preneoplastic and neoplastic ECL-cell proliferations.^201^ MENIN immunohistochemistry is not of help for the diagnosis, since *MEN1* mutations and loss of heterozygosity can also occur in 17% to 73% of type 1 ECL-cell NETs.^206^

**FIGURE 14:**
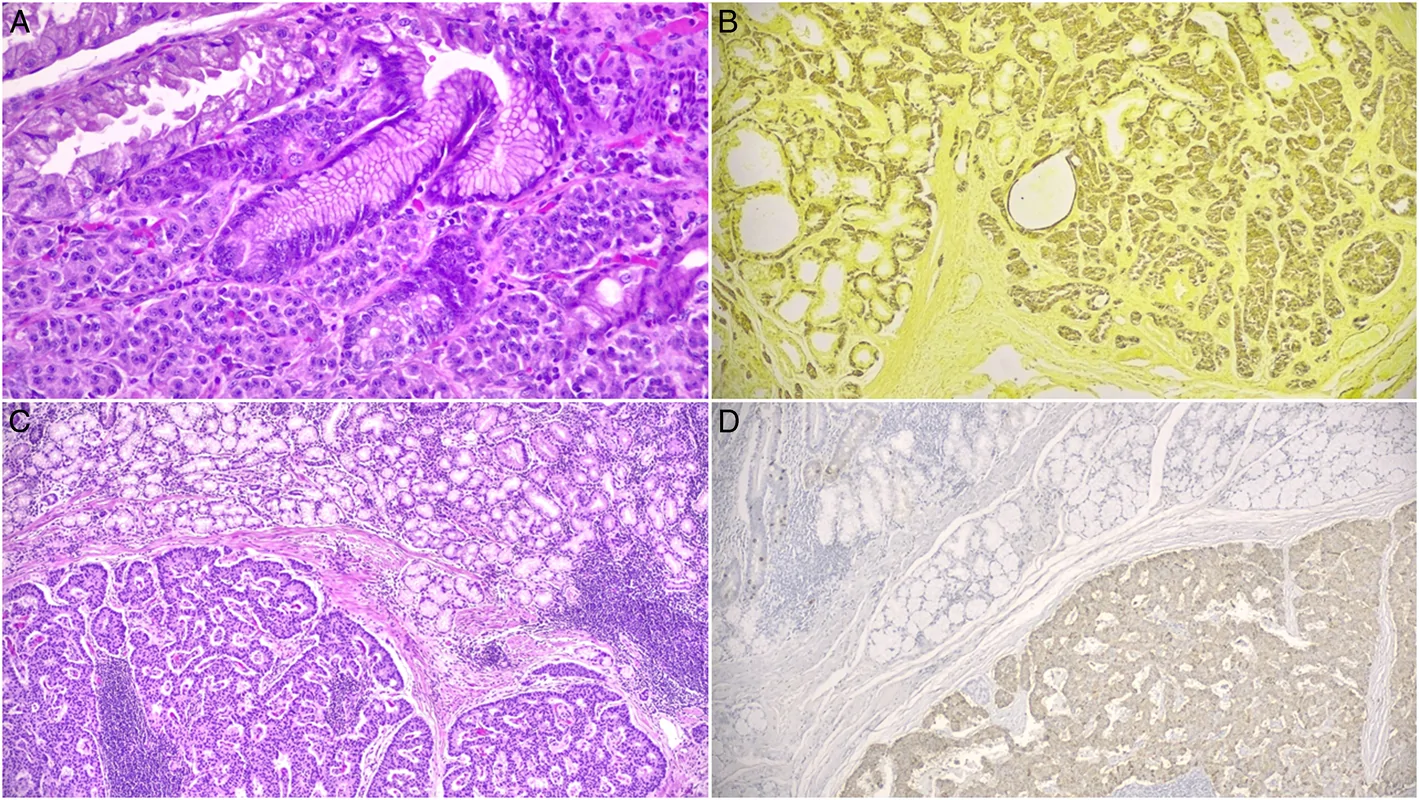
ECL-cell tumor of type 2 is characterized by a proliferation of monomorphic, well-differentiated neuroendocrine cells. A few nonatrophic peritumoral glands are observed (A). Grimelius’ silver stain (B) is positive in the tumor (right part of the image) and shows linear and micronodular ECL-cell hyperplasia in the peritumoral mucosa (left part of the image). The patient had MEN1 syndrome and presented with a duodenal gastrinoma (C) composed of gastrin-positive cells (D).

The 5-year overall survival rate of type 2 ECL-cell NETs ranges from 60% to 90%.^207^ The prognosis largely depends on the management of the gastrinoma-related Zollinger-Ellison syndrome, although lymph node and distant metastases from the gastric tumor are observed in about 20% and 10% of patients, respectively.^202,204,208,209^

Gastric NETs bearing *CDKN1B* mutation with reduced P27^Kip1^ expression have been described in the setting of *MEN4* syndrome.^210^

### Duodenum and Ampulla of Vater

Duodenal/ampullary NETs are rare and mostly nonfunctioning, although the duodenum may be considered as a “hormone hypersecretion syndrome-enriched site” with particular reference to gastrinomas.^211^ In a recent study by our group, it was demonstrated that, independent of the clinical hypersecretion syndrome, the immunohistochemical typing of tumor cells according to hormone and transcription factor expression is an important parameter in determining tumor aggressiveness.^212^ A small but well-characterized proportion of duodenal/ampullary NETs arises in the context of an inherited cancer-predisposing syndrome, mainly MEN1 and NF1.

About 10% of duodenal/ampullary NETs arise in the context of MEN1 syndrome, mainly presenting as multiple gastrinomas, associated with excessive gastric acid secretion, multiple peptic ulcers, diarrhea, and gastroesophageal reflux (Zollinger-Ellison syndrome, ZES).^213^ In patients with ZES in MEN1, large periduodenal lymph node metastases may be detected at abdominal imaging, whereas small duodenal/ampullary primaries may be difficult to identify.^214^ Very rare somatostatin-producing NETs have been described in the context of MEN-1 syndrome.^215^ The histopathologic features of MEN1 syndrome-associated duodenal and ampullary tumors are largely similar to those of their sporadic counterparts. However, the presence of multiple lesions and the identification of precursor changes, such as hyperplasia of G- and D-cells in the duodenal mucosa, are important clues suggesting an underlying genetic predisposition.^179^

Around 10% of patients with NF1 develop a somatostatin-secreting tumor. These NETs are usually nonfunctional and are virtually always located in the ampullary region^216^ and, in turn, account for about 20% of all somatostatin-secreting tumors at this site.^152,217,218^ At the genomic level, in addition to *NF1* biallelic inactivation, a recurrent alteration of this neoplasm regards the deletion/loss of heterozygosity of chromosome 22.^153^ The histopathologic appearance of somatostatin-secreting NETs in NF1 is similar to that of sporadic tumors, showing a tubule-glandular pattern with intraluminal psammoma bodies that, in NF1 cases, seem to be more numerous and, rarely, mucin-like material (Fig. 15).^219^ These patterns of growth may sometimes be misinterpreted as a low-grade adenocarcinoma. Careful morphologic observation and immunohistochemical stains with general neuroendocrine markers and somatostatin are crucial for the correct diagnosis.

**FIGURE 15:**
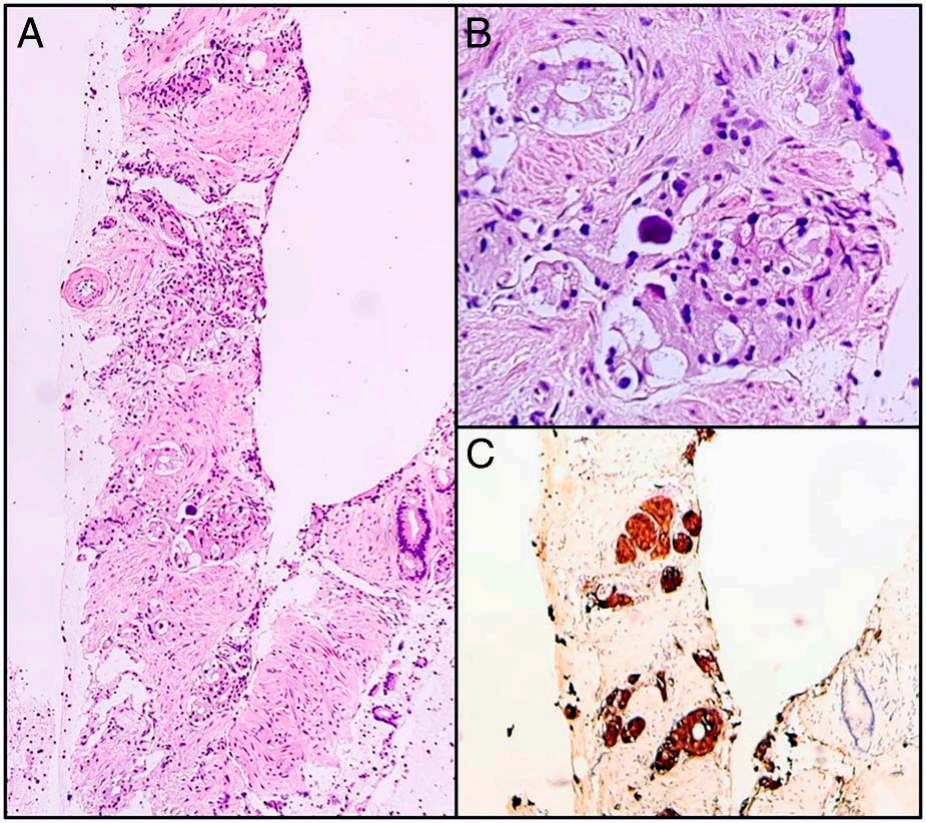
Ampullary somatostatin-producing tumors are typically found in patients with NF1, are characterized by infiltrative growth, and are composed of well-differentiated cells that form pseudoglandular structures (A). At a higher magnification, a psammomatous body is clearly visible (B). Tumor cells are strongly and diffusely immunoreactive for somatostatin (C).

### Distal Small Intestine

Jejunal-ileal NETs account for 40% of malignancies at this site and are sporadic in most cases. Scandinavian studies have demonstrated an increased relative risk for these tumors in first-degree relatives.^220–222^ The existence of a hereditary predisposition has been more recently confirmed also in second- and third-degree relatives in epidemiological studies,^223^ and the presence of at least 2 affected members in a family defines a familial jejunal-ileal NET. Nevertheless, the search for the genetic driver of familial NETs in the jejunum/ileum has not yet yielded definitive results.^224^ In general, the genetic and molecular landscape of jejunal-ileal NETs does not provide significant clues to a possible heritable abnormality. Indeed, these tumors have been reported to bear the lowest tumor mutational burden among human malignancies, and recurrent gene mutations are never detected.^3,225^ A few studies have proposed candidate gene mutations in *IPMK* (inositol polyphosphate multikinase gene),^226^ *MUTYH* and *OGG1*,^227,228^ and chromosome 18 alterations, but none of these have been confirmed on large-scale studies.^229,230^

### Rectum

A subset of colo-rectal neuroendocrine carcinomas is known to be associated with hereditary syndromes related to colorectal cancer (familial adenomatous polyposis, Lynch syndrome, MYH-associated polyposis, and CHEK2-related cancer syndrome), which, however, only exceptionally involve rectal NETs.^231^ “Classic” syndromes associated with NETs (MEN1, VHL, NF1, and TSC) do not typically involve rectal NETs. However, a case of multiple rectal NETs in a patient with NF1 and a case of rectal L-cell NET in a patient with TSC have been reported.^232,233^

## CONCLUDING REMARKS

Contemporary pathology provides a natural bridge between morphology, clinical information, and genetics. In the context of hereditary neuroendocrine tumors, the pathologist plays a pivotal role in identifying distinctive morphologic features of neoplastic proliferations and recognizing associated preneoplastic lesions. Immunohistochemistry and somatic molecular testing may support the suspicion of an inherited condition, help confirm it, and in some cases provide a rationale for germline genetic testing. Continuous interaction within a multidisciplinary team ensures a bidirectional flow of information, which is essential for optimal patient management.
